# Next-generation sequencing identifies unexpected genotype-phenotype correlations in patients with retinitis pigmentosa

**DOI:** 10.1371/journal.pone.0207958

**Published:** 2018-12-13

**Authors:** Johannes Birtel, Martin Gliem, Elisabeth Mangold, Philipp L. Müller, Frank G. Holz, Christine Neuhaus, Steffen Lenzner, Diana Zahnleiter, Christian Betz, Tobias Eisenberger, Hanno J. Bolz, Peter Charbel Issa

**Affiliations:** 1 Department of Ophthalmology, University of Bonn, Bonn, Germany; 2 Center for Rare Diseases Bonn (ZSEB), University of Bonn, Bonn, Germany; 3 Institute of Human Genetics, University of Bonn, Bonn, Germany; 4 Bioscientia Center for Human Genetics, Ingelheim, Germany; 5 Institute of Human Genetics, University Hospital of Cologne, Cologne, Germany; 6 Oxford Eye Hospital, Oxford University Hospitals NHS Foundation Trust, and Nuffield Laboratory of Ophthalmology, Department of Clinical Neurosciences, University of Oxford, Oxford, United Kingdom; University of Florida, UNITED STATES

## Abstract

Retinitis pigmentosa (RP) is an inherited degenerative disease causing severe retinal dystrophy and visual impairment mainly with onset in infancy or adolescence. Targeted next-generation sequencing (NGS) has become an efficient tool to encounter the enormous genetic heterogeneity of diverse retinal dystrophies, including RP. To identify disease-causing mutations in unselected, consecutive RP patients, we conducted Sanger sequencing of genes commonly involved in the suspected genetic RP subtype, followed by targeted large-panel NGS if no mutation was identified, or NGS as primary analysis. A high (70%) detection rate of disease-causing mutations was achieved in a large cohort of 116 unrelated patients. About half (48%) of the solved RP cases were explained by mutations in four genes: *RPGR*, *EYS*, *PRPF31* and *USH2A*. Overall, 110 different mutations distributed across 30 different genes were detected, and 46 of these mutations were novel. A molecular diagnosis was achieved in the majority (82–100%) of patients if the family history was suggestive for a particular mode of inheritance, but only in 60% in cases of sporadic RP. The diagnostic potential of extensive molecular analysis in a routine setting is also illustrated by the identification of unexpected genotype-phenotype correlations for RP patients with mutations in *CRX*, *CEP290*, *RPGRIP1*, *MFSD8*. Furthermore, we identified numerous mutations in autosomal dominant (*PRPF31*, *PRPH2*, *CRX*) and X-linked (*RPGR*) RP genes in patients with sporadic RP. Variants in *RP2* and *RPGR* were also found in female RP patients with apparently sporadic or dominant disease. In summary, this study demonstrates that massively parallel sequencing of all known retinal dystrophy genes is a valuable diagnostic approach for RP patients.

## Introduction

Retinitis pigmentosa (RP) is one of the most common forms of inherited retinal degenerations, affecting about 1:3.000 individuals.[1, 2] It is characterized by a primary rod photoreceptor degeneration and consecutive cone photoreceptor death, leading to night blindness and subsequent progressive vision loss. Legal blindness at working age is common in RP patients, with devastating professional and social implications for the patients.[1, 2] Although typical characteristics of RP have been described, there is broad phenotypic variability, regarding both clinical presentation and age of onset. RP is genetically heterogeneous, with more than 60 disease-causing RP genes reported so far (RetNet, https://sph.uth.edu/retnet/). With novel upcoming therapeutic options such as gene therapy, the identification of the disease-causing mutations has gained importance.[3]

Diagnostic genotyping in RP has become very efficient with the implementation of next-generation sequencing (NGS),[4–7] enabling parallel sequencing of all known RP (and, if needed, many more) genes which improves our understanding of the disorder’s pathogenesis.

Here, we report unexpected molecular findings and novel genotype-phenotype correlations in a cohort of 116 consecutive and unrelated patients seen in a retinal dystrophy clinic of a German tertiary referral center. In addition, the application of targeted NGS of all known RP genes provides insight into the mutational spectrum of this representative cohort.

## Methods

### Patients

This retrospective single-center cross-sectional study included 116 consecutive and unrelated patients who were investigated at the Department of Ophthalmology, University of Bonn, Germany. After clinical diagnosis of RP, molecular screening was performed. The study was in adherence with the declaration of Helsinki. Institutional review board approval (Ethics Committee, Medical Faculty, University of Bonn, Germany) and patients' informed consent were obtained. All patients underwent genetic counseling, which included family history assessment. Asymptomatic relatives of index patients received genetic counselling prior to mutation testing.

### Image acquisition and functional testing

Clinical assessment included standardized anterior segment and dilated fundus examination, best corrected visual acuity (BCVA), and visual field testing (30–2 threshold program; HFA II; Carl Zeiss Meditec, Dublin, CA, USA), and, in selected cases, electroretinography (ERG). Retinal imaging consisted of spectral domain optical coherence tomography (OCT), fundus autofluorescence (AF) imaging (both, Spectralis HRA+OCT, Heidelberg Engineering, Heidelberg, Germany), fundus photography (Zeiss, Visucam, Oberkochen, Germany) and wide-field fundus imaging (Optos PLC, Dunfermline, United Kingdom).

### Molecular genetic analysis

Genomic DNA was extracted from blood lymphocytes by a standard protocol. A two-tier procedure was implemented for patients suggestive for autosomal recessive or sporadic RP. A step-wise molecular screening was chosen, with initial Sanger sequencing of genes commonly involved in the pathogenesis of the suspected genetic RP subtype (*EYS* and *RP1* for autosomal recessive RP, *PRPF31* for autosomal dominant RP).[8] If no mutation was identified in this initial sequencing of singular genes, targeted NGS on an Illumina Hiseq1500 system was carried out for the RP genes known at the respective time of analysis (see S1 Table) after enrichment using NimbleGen sequence capture technology (as described previously[8]). Importantly, the NGS panels also contained the genes for clinically overlapping conditions such as cone/cone-rod dystrophies, Leber’s congenital amaurosis (LCA) and syndromes with retinal dystrophies, allowing for an extended genetic assessment if no mutation was found in genes previously associated with RP, without need for additional experimental efforts. To detect X-linked RP-causing mutations, we added NGS of amplicons comprising *RPGR*_*ORF15*_ to panel-NGS of the remaining exons of *RPGR* and *RP2*. Based on our step-wise molecular screening, Sanger sequencing and targeted NGS identified disease-causing mutations in 20 and 61 cases, respectively. Verification of mutations identified in NGS and segregation analyses were carried out by PCR and subsequent Sanger sequencing.

To determine the most likely inheritance mode, pedigrees were generated based on the patients’ family history including at least three generations. Autosomal recessive inheritance was assumed in case of parental consanguinity and/or if only siblings were affected. Autosomal dominant inheritance was assumed if there was a positive family history for at least three successive generations, and likely autosomal dominant if different but not successive generations were affected in absence of known consanguinity (e.g., assuming reduced penetrance, or because a linking individual died early or lost contact). X-linked inheritance was assumed in families with only males being severely affected and at least considered if male-to-male transmission was lacking in families with RP patients in different generations. RP was categorized as sporadic in case of negative family history. If no family history was available, e.g. because the patient was adopted and without contact with his biological parents, no candidate inheritance mode was defined.

Variants were filtered against dbNSFP v2.0, dbSNP v137, gnomAD (exomes) and the Human Gene Mutation Database (HGMD Professional 2017.3). The cut-off for the maximum minor allele frequency (MAF) was set to 1%.[9] Nonsense, frameshift, large deletions and canonical splice site variants were regarded pathogenic. Rare non-synonymous single nucleotide variations were considered likely pathogenic when at least half of the algorithms of used in silico prediction software tools predicted that the variant is probably damaging and when it was predicted as conserved with conservation prediction algorithms. Functional predictions were carried out using SIFT, PolyPhen2, MutationTaster, MutationAssessor, FATHMM, LRT, VEST, CADD, PROVEAN and DANN. Splice sites were predicted with AdaBoost and RF. Assessment of conservation was done by PhyloP, GERP++, PhastCons, SiPhy, Grantham Distance and BLOSUM62.

## Results and discussion

**Fig 1:**
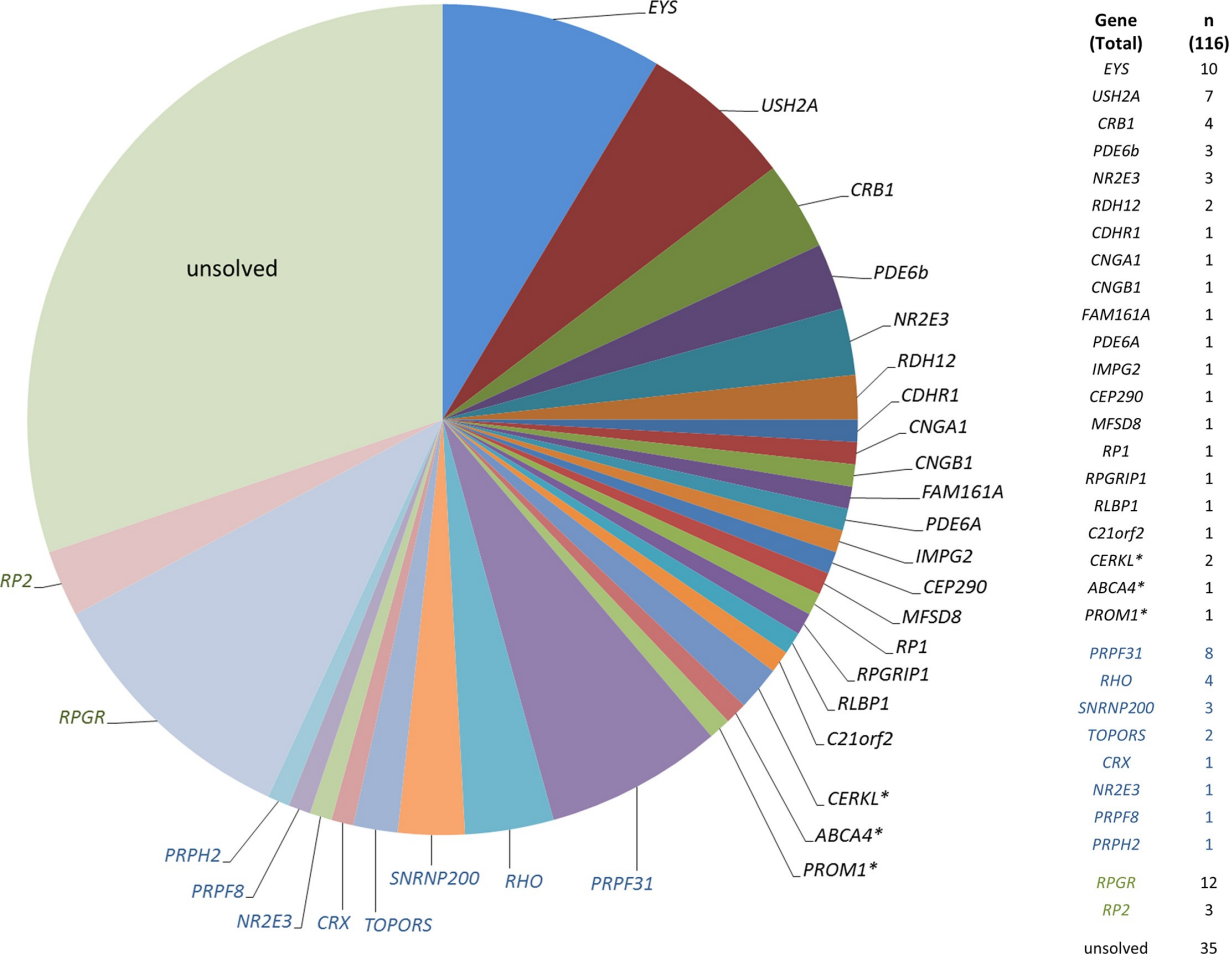
Distribution of mutations in consecutive, unrelated RP patients. Genes mutated in patients who were reversely phenotyped (based on the genetic findings) are indicated by asterisk. The number of patients with mutations in the different genes is displayed on the right. Black, autosomal recessive. Blue, autosomal dominant. Green, X-linked.

Approximately 99% of all coding exons were covered at least 20-fold. Disease-causing mutations were identified in 81 (70%) out of the 116 analyzed patients, a high diagnostic yield compared to previous reports using targeted NGS in RP patients (S2 and S3 Tables).[4–8, 10–20]. The wide range of reported diagnostic yields in cohorts >50 patients (25%-80%)[4, 7, 8, 10–17] may be due to differences in cohort sizes, populations, inclusion criteria, differences in clinical assessment, NGS platforms, and bioinformatic pipelines. Cases without mutations in the genes analysed herein can be due to causative variants in non-coding regions of these genes or in genes not yet known to underlie retinal degeneration, or–less likely–due to mutations in regions below optimal coverage. Overall, 110 different mutations distributed across 30 different genes were detected in this study, and 46 of these mutations were novel at the time of molecular diagnosis (Fig 1, S2 and S4 Tables).

The clinical classification was revised in four patients who had variants in genes usually associated with an early macular degeneration (*CERKL*, *PROM1* and *ABCA4*). They presented with very late disease stages, preventing a clear classification of the initial phenotype as cone-rod or rod-cone dystrophy. However, re-evaluations of these patients’ histories were compatible with retinal dystrophies that initially affected the central retina and/or the cone system. Classification may be particularly difficult in patients with *CERKL* mutations, as their initial symptoms may manifest in dim light conditions, although their retinal phenotype may indicate a dystrophy primarily affecting the central retina.[21] Thus, a combination of comprehensive clinical phenotyping and molecular testing improves the accuracy of diagnoses in genetically and clinically heterogeneous diseases.

About half (48%) of the remaining 77 solved RP cases were explained by mutations in four genes: *RPGR* (n = 12, 16%), *EYS* (n = 10, 13%), *PRPF31* (n = 8, 10%) and *USH2A* (n = 7, 9%). Based on the genetic findings, inheritance turned out to be autosomal recessive in 56% (n = 45), autosomal dominant in 26% (n = 21), and X-linked in 19% (n = 15) of patients. If the family history was highly suggestive for a particular mode of inheritance (n = 45), a molecular diagnosis was achieved in 82–100%, then confirming the assumed inheritance (Table 1). The mutation detection rate was lower (60%; 42 out of 70 patients) in cases of sporadic RP (see below), similar to a previous reported cohort of patients with macular and cone/cone-rod dystrophies.[22]

**Table 1:** More of inheritance based on family history, on genetic results, and mutation detection rate for each group.

Assumed inheritance based on family history	n	Inheritance based on mutation	n	Mutation detection rate %
Autosomal recessive	24	arRP	20	83%
Autosomal dominant (n = 15) or likely autosomal dominant (n = 2)	17	adRP	13	82%
		XLRP	1	
X-linked	4	XLRP	4	100%
Unclassified	1	adRP	1	100%
Sporadic	70	arRP	25	60%
		adRP	7	
		XLRP	10	

In the following, unexpected findings identified in 21 (27%) out of the 77 solved RP patients are described in detail (variants displayed in Table 2). These include a high proportion of autosomal dominant or X-linked mutations in patients with sporadic RP, novel or uncommon genotype-phenotype correlations (with no mutations in genes knowingly associated with the respective phenotype), and X-linked mutations in females with apparently sporadic or dominant RP.

**Table 2:** Molecular findings in patients with unexpected genotyping results.

ID (#)	Panel	Gender (m/f)	Gene	Zygosity	Exon/Intron (IVS)	Nucleotide	Protein	Segregation analysis	Reference
36	II	f	*CEP290*	Heterozygous	Exon 12 Intron 26	c.982C>T c.2991+1655A>G	p.Gln328* splice site/p.Cys998*	yes	novel [8, 23]
37	II	m	*MFSD8*	Homozygous	Exon 13	c.1445G>C	p.Arg482Pro	yes	novel
39	II	m	*RPGRIP1*	Homozygous	Exon 19	c.3100_3238del139	p.Gln1034Thrfs*23	no	novel
46	I	f	*PRPF31*	Heterozygous	Exon 8	c.839T>G	p.Val280Gly	yes	novel
47	II	m	*PRPF31*	Heterozygous	Exon 1–3	deletion of exons 1–3	deletion of exons 1–3	yes	[8, 24]
48	III	f	*PRPF31*	Heterozygous	Intron 7	c.698-1G>A	splice	no	[25]
49	I	f	*PRPF31*	Heterozygous	Exon 1–5	deletion of exons 1–5	deletion of exons 1–5	yes	[8, 24]
50	Sanger	m	*PRPF31*	Heterozygous	Exon 8	c.816_830delCTACATCTACCACAG	p.Tyr273_Ser277del	no	novel
63	III	m	*CRX*	Heterozygous	Exon 3	c.122G>A	p.Arg41Gln	yes	[26]
66	IV	f	*PRPH2*	Heterozygous	Exon 1	c.422A>G	p.Tyr141Cys	no	[27]
68	III	f	*RPGR*	Heterozygous	ORF15	c.2442_2445del AGAG	p.Gly817Lysfs*2	no	[28]
69	ORF15-Amplicon	m	*RPGR*	Hemizygous	ORF15	c.2452G>T	p.Glu818*	no	novel
71	IV	m	*RPGR*	Hemizygous	Exon 8	c.917A>C	p.His306Pro	no	novel
72	ORF15-Amplicon	m	*RPGR*	Hemizygous	ORF15	c.2630delA	p.Glu877Glyfs*212	yes	novel
73	IV	m	*RPGR*	Hemizygous	Exon 9	c.1006A>T	p.Asn336Tyr	no	novel
74	ORF15-Amplicon	m	*RPGR*	Hemizygous	ORF15	c.2426_2427delAG	p.Glu809Glyfs*25	no	[28]
75	III	f	*RPGR*	Heterozygous	ORF15	c.2405_2406delAG	p.Glu802Glyfs*32	no	[28]
76	IV	m	*RPGR*	Hemizygous	ORF15	c.3034delG	p.Glu1012Lysfs*77	no	novel
77	V	m	*RPGR*	Hemizygous	Exon 3	c.194G>T	p.Gly65Val	no	[29–31]
79	III	f	*RP2*	Heterozygous	Exon 3	c.829dupG	p.Ala277Glyfs*11	no	novel
80	I	m	*RP2*	Hemizygous (47,XXY karyotype, skewed X inactivation)	Exon 2	c.630_633delTCGT	p.Arg211Phefs*26	no	novel

### High proportion of autosomal dominant and X-linked inheritance in sporadic RP

In the majority of patients with sporadic RP, the retinopathy is autosomal recessively inherited, usually with a small recurrence risk in the offspring. Although this was confirmed by our findings, it is noteworthy that X-linked (n = 10) and autosomal dominant (n = 7) mutations accounted for disease in 24% of the sporadic cases (Table 1).

Similar rates of X-linked RP have been reported for overall sporadic (4–6%,[10, 32]) or for male sporadic cases (15%-30%[33, 34]). Of note, two sporadic female patients with X-linked RP were identified in this study (see below). Most autosomal dominant mutations in sporadic RP patients were identified in *PRPF31* (n = 5, #46–50 in S2 Table). Only one patient carried a *CRX* mutation (see below, patient #63), and one patient had a previously described assumingly pathogenic variant in *PRPH2* (c.422A>G, p.Tyr141Cys; patient #67; variant not carried by her mother, no further family members were available for segregation analysis). Previously it has been shown that mutations in *PRPH2* cause highly variable and mild RP phenotypes, and index patients may be erroneously classified as simplex cases because of undiagnosed family members with mild disease.[35] Incomplete penetrance of *PRPF31*-associated RP is a well-documented phenomenon.[36] Our study underlines the high rate of non-penetrance in *PRPF31* mutations and/or that the family history of relatively small pedigrees is less reliable in identifying such low- or non-penetrant dominant mutations.[36, 37] Segregation analysis for *PRPF31* mutations was possible in three families, revealing a *de novo* mutation in one patient (#47, S2 Table). Healthy individuals with *PRPF31* mutations were fully examined and showed no morphological fundus changes (including peripheral AF recordings). Of note, they had slightly reduced or borderline low responses on scotopic ERG testing, indicating that *PRPF31* mutation carriers may exhibit reduced retinal function at subclinical level.[38, 39]

### RP caused by a *CRX* mutation previously associated with cone-rod dystrophy

**Fig 2:**
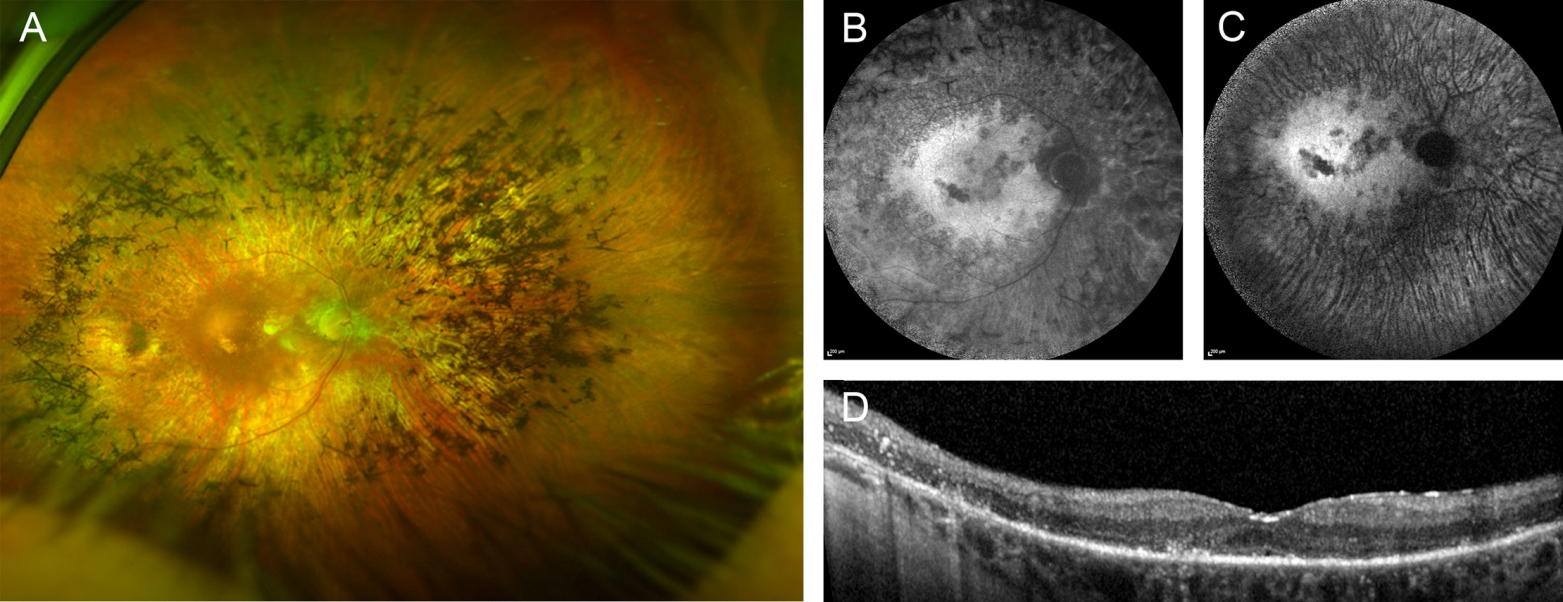
Right eye of a patient with RP due to an *CRX* mutation. (A) widefield false-color image, fundus AF with (B) 488 nm and (C) 787 nm excitation light, and (D) spectral-domain optical coherence tomography. Only one eye is shown due to high symmetry between eyes.

An 83-year old patient (#63, S2 Table). without visual limitations throughout his professional life reported impaired dark adaption, nyctalopia and glare as first symptoms in his early 8^th^ decade of life. Funduscopy showed typical RP fundus changes (Fig 2). He also noted progressive loss of visual acuity over the past 7 years, and visual acuity was now 20/200 in the right eye and hand movements on the left eye. The visual field was severely constricted, and ERG responses were not detectable. Although there was no known affected family member, exclusion of a dominant inheritance of this late onset RP was not possible because most of his relatives died at relatively young age. Genetic testing showed a heterozygous missense mutation (c.122G>A, p.Arg41Gln) in exon 3 of the *CRX* gene. The same mutation had previously been described in families with autosomal dominant late-onset cone-rod dystrophy,[26] indicating a novel genotype-phenotype correlation for this particular mutation.

### Non-syndromic RP caused by mutations in *CEP290*

**Fig 3:**
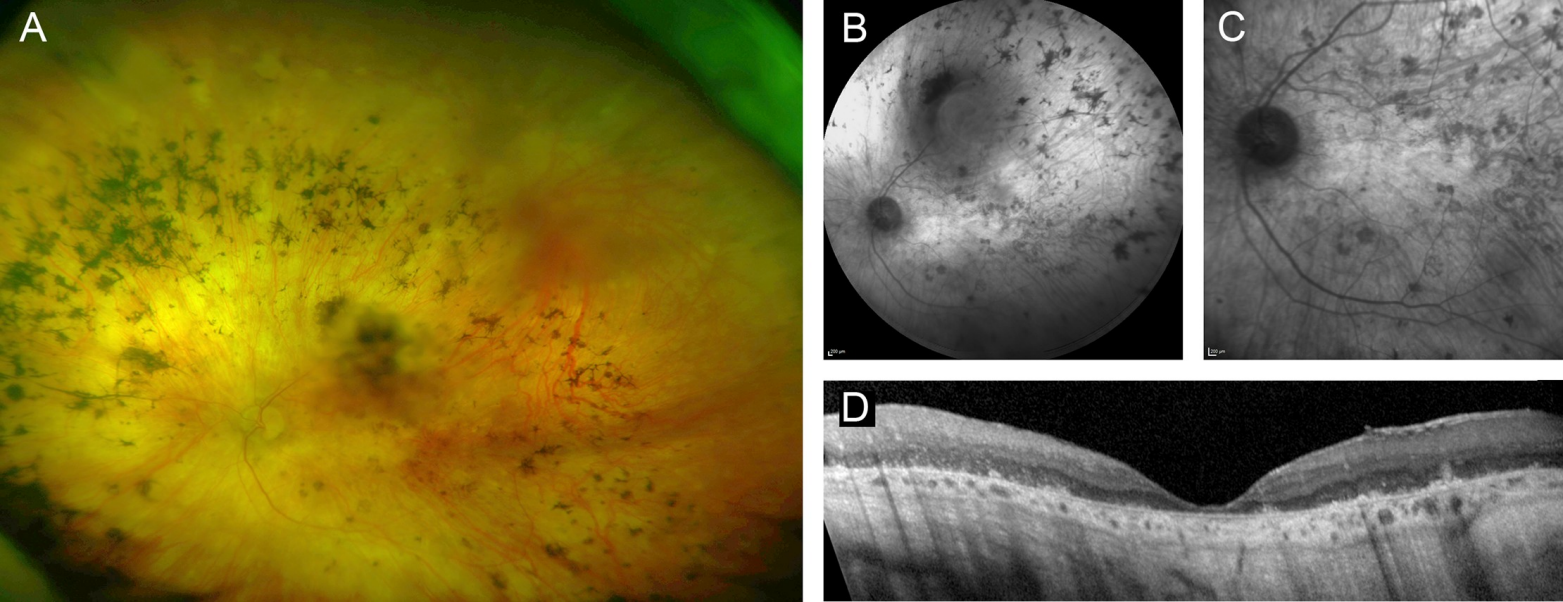
Left fundus of a 70-year-old women with RP caused by compound-heterozygous *CEP290* mutations. (A) widefield fundus imaging, (B, C) near infrared reflectance imaging, (D) spectral-domain optical coherence tomography. Only one eye is shown due to high symmetry between eyes.

A female patient (#36, S2 Table) with reduced night vision and dark adaption since childhood had graduated from university without visual aids. Starting in her mid to late twenties, she had noticed progressive vision loss and was diagnosed with RP at the age of 27. Now 70 years-old, visual acuity was reduced to light perception in both eyes. Clinical examination showed widespread atrophy of the outer retina and bone-spicule pigmentations (Fig 3). Targeted NGS revealed heterozygosity for a nonsense mutation (c.982C>T, p.Gln328*) in exon 12 and a deep-intronic mutation (c.2991+1655A>G) in intron 26 that creates a strong splice-donor site in *CEP290* and a premature stop codon, p.Cys998*. Compound-heterozygosity was deduced because the patient’s son carried the nonsense, but not the splice site mutation.

Biallelic mutations in *CEP290* cause several syndromic ciliopathies and non-syndromic LCA.[13, 23, 40–42] Of note, our patient had no abnormalities in motor or cognitive development, renal cysts or other morphological abnormalities indicating a *CEP290*-associated syndrome. Although an association of *CEP290* mutations with relatively milder non-syndromic retinal phenotypes has been described in a few previous reports, vision loss in those patients either occurred in the first decade of life or the history of vision loss was not described in detail.[4, 7, 8, 16, 43, 44] Only one recent comparable study reported a patient with an apparently similar disease course.[45] The latter and our case also show that retinal disease due to *CEP290* mutations is not necessarily associated with poor cone function,[46] since both patients had normal visual acuity apart from reduced night vision for at least the first two decades of life. It is likely that the phenotypic continuum of *CEP290*-related non-syndromic retinal dystrophies can be attributed to modifying retina-relevant variants elsewhere in the genome. The identification of *CEP290*-related RP, a second non-syndromic phenotype associated with mutations in this gene, further supports the categorization of Joubert syndrome (JBTS) genes as strong candidates for isolated retinopathies. *OFD1* and, very recently, *AHI1*, have also been shown to cause both JBTS and isolated retinal degeneration.[47, 48]

### Non-syndromic RP caused by *RPGRIP1* mutations

**Fig 4:**
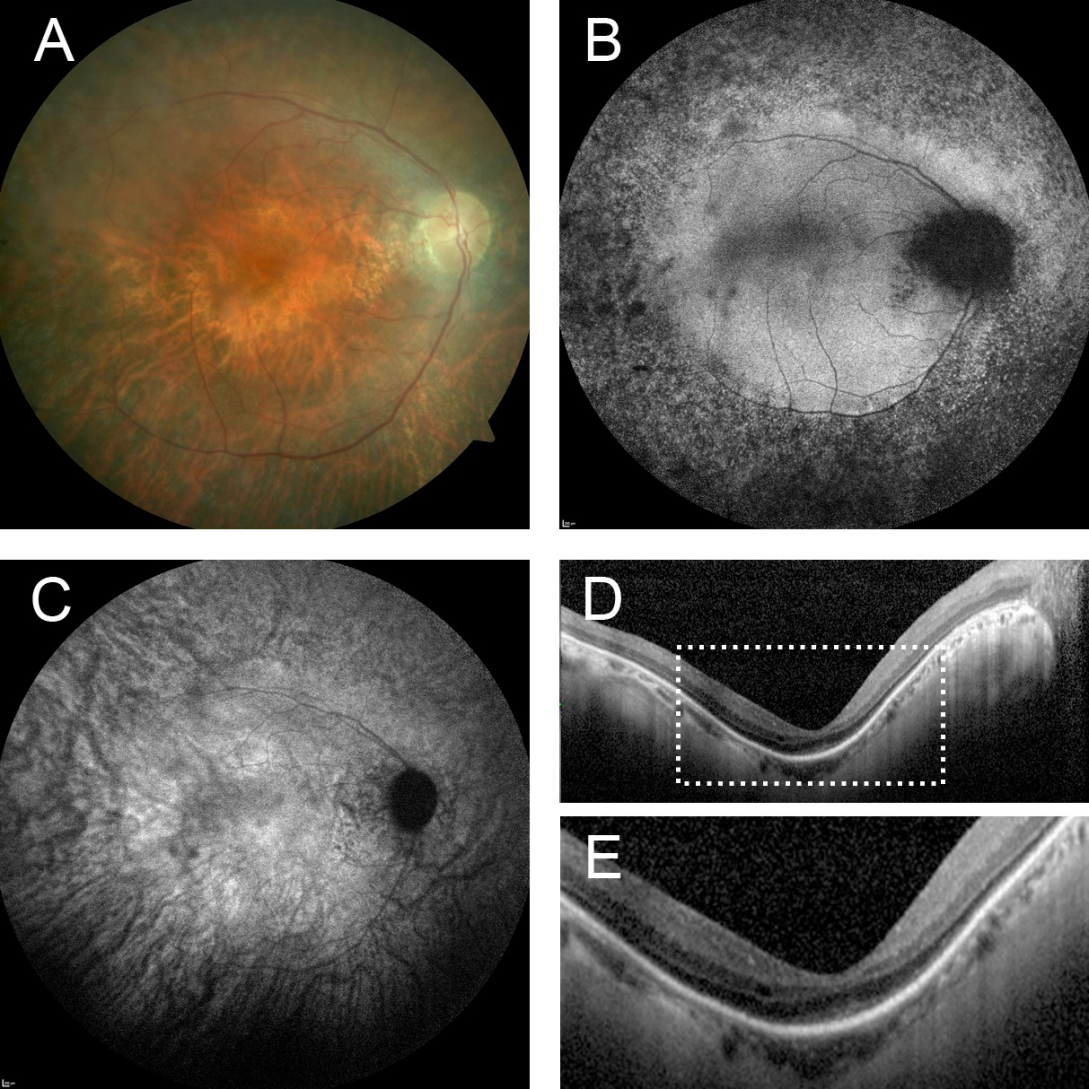
Right eye of a patient with RP and a homozygous *RPGRIP1* mutation. Fundus color image (A), fundus AF with (B) 488 nm and (C) 787 nm excitation light, (D, E) spectral-domain optical coherence tomography. Only one eye is shown due to high symmetry between eyes.

A 42-year-old male patient (#39, S2 Table) was diagnosed with sporadic RP at the age of 13 years. He reported nyctalopia, dark adaption problems and glare since childhood. At the age of 37 years, he started using visual aids. He underwent cataract surgery, and visual acuity now was 20/50 in the right and 20/63 in the left eye. Visual fields were severely constricted and full-field ERG showed no detectable responses. Funduscopy showed alterations characteristic for RP, with widespread retinal pigment epithelial and photoreceptor atrophy, and bone spicule pigmentations (Fig 4). Targeted NGS revealed a novel homozygous deletion (c.3100_3238del139, p.Gln1034Thrfs*23) of exon 19 of *RPGRIP1*, resulting in a frameshift predicted to result in unstable mRNA or protein truncation.

Mutations in *RPGRIP* are a known cause of LCA [42, 49, 50] and juvenile RP.[51–53] Besides its uncommon benign disease course, the presented case is exceptional because visual acuity was good in early life and remained relatively stable until an age beyond 40 years. This contrasts with previous observations which had suggested severe cone functional loss despite relative sparing of the fovea anatomy on OCT images.[53]

### Non-syndromic RP caused by mutations in *MFSD8*

**Fig 5:**
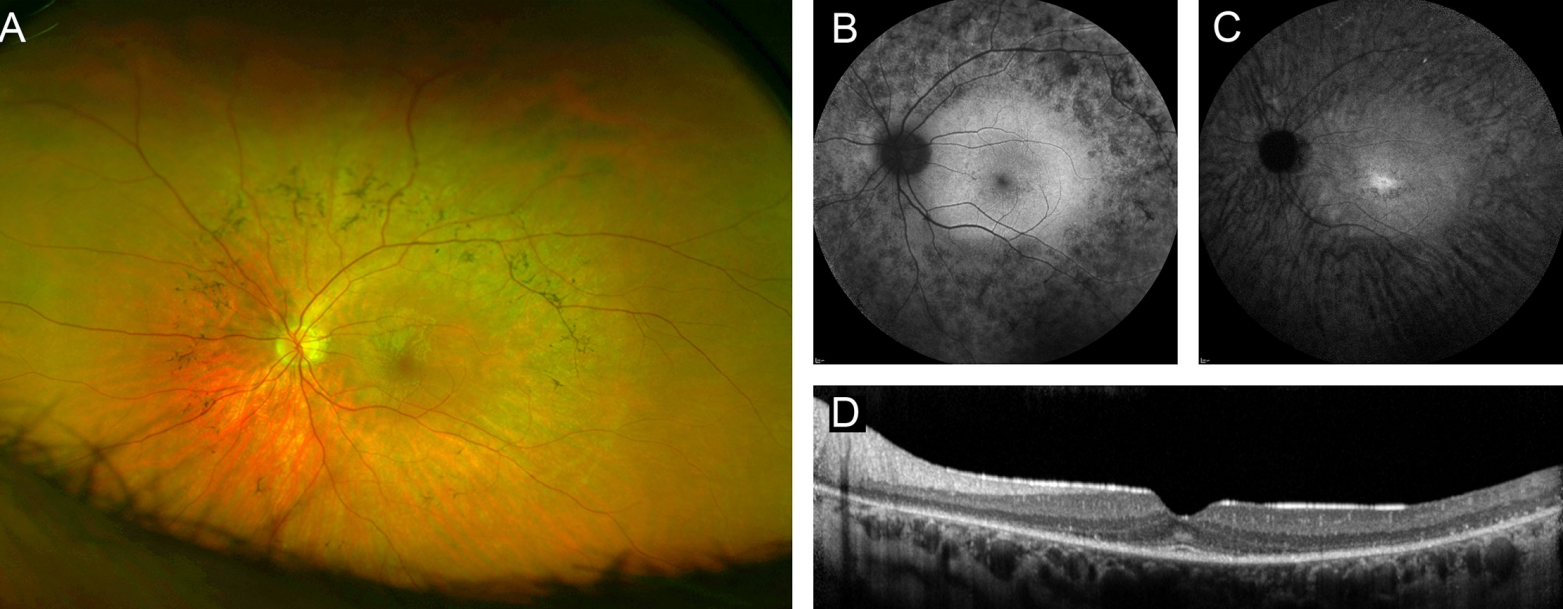
Left eye of a patient with RP and a homozygous *MFSD8* mutation. (A) widefield false-color image, fundus AF with (B) 488 nm and (C) 787 nm excitation light, (D) spectral-domain optical coherence tomography. Only one eye is shown because there was high similarity of both eyes.

A 35-year old patient (#37, S2 Table) who initially noticed nyctalopia, dark adaption problems and glare at the age of 31 years showed fundus changes typical for RP (Fig 5). Best corrected visual acuity was 20/40 and 20/25, and there was severe concentric constriction of the visual fields. ERG showed no detectable responses. Genetic testing identified a homozygous variant (c.1445G>C, p.Arg482Pro) in exon 13 in the *MFSD8* gene. *In silico* assessment with various programs strongly supports the categorization as disease-causing mutation, as did the results from segregation analysis: The index patient’s likewise affected brother and a second brother who reported difficulties seeing in dim light (not examined) carried the *MFSD8* missense mutation in homozygous state, whereas a sister with normal vision carried the wild-type allele.

*MFSD8* mutations are known to cause variant late-infantile neuronal ceroid lipofuscinosis (vLINCL; CLN7), an early-onset severe lysosomal storage disorder with intralysosomal accumulation of autofluorescent lipopigments, presenting with seizures, mental regression and retinopathy, which was either not further specified [54–58] or suggestive for RP.[59] Recently, *MFSD8* mutations have been described in families with non-syndromic autosomal recessive macular dystrophy with central cone involvement, isolated maculopathy and generalized retinopathy.[60, 61] Similarly, the patient in our study showed no neurologic features typical for vLINCL, confirming *MFSD8* as a non-syndromic retinopathy gene. The *MFSD8* genotype-phenotype correlation proposed earlier,[61] with vLINCL resulting if both mutations are severe (in particular truncating mutations), whereas milder mutations (such as hypomorphic missense mutations) on at least one gene copy result in non-syndromic retinal degeneration, likely also applies in the family reported herein.

### *RP2* and *RPGR* mutations in female patients with apparently sporadic or dominant RP

**Fig 6:**
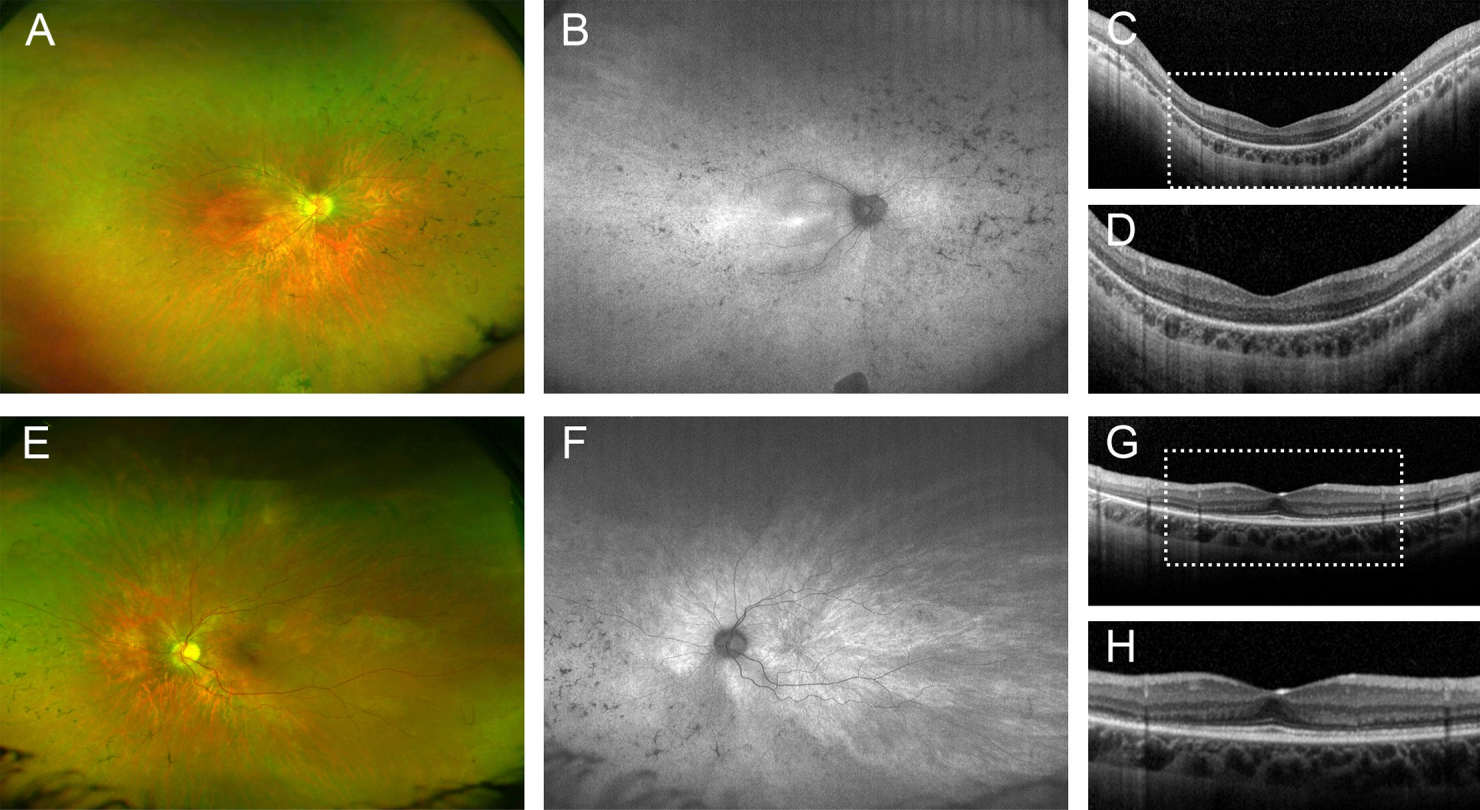
Phenotype of an RP patient with an *RP2* mutation. A-D right eye, E-H left eye Right eye: (A, E) widefield fundus imaging and (B, F) widefield fundus autofluorescence, (C, D, G, H) spectral-domain optical coherence tomography.

Patient A (RP2): In this 16-years-old young woman (#80, S2 Table), unilateral reduced vision was first recognized at the age of four years, but no further examinations had been initiated at that time. When examined at the age of 12 years, she reported impaired central vision, but no nyctalopia. Visual acuity in the right and left eye was 20/63 and 20/20, respectively. The right eye was emmetropic and the left eye was myopic (spherical equivalent -3,5 dpt). The visual fields were severely constricted in the right eye, and there was a nasal superior visual field loss in the left eye. The ERG showed extinct rod responses in her right eye, while responses in her left eye were severely reduced. When she was examined at the age of 16, visual acuity had deteriorated only in the right eye (now 20/100). Fundus examination revealed narrowed vessels, outer retinal atrophy and bone spicule pigmentations, all much more pronounced in the right eye. In addition, the left eye showed a tapetal-like reflex (Fig 6). Fundus AF confirmed the asymmetry and revealed a pattern of radial lines extending into the fundus periphery in the left eye, which is a characteristic finding in carriers of X-linked RP. NGS analysis identified a one base-pair duplication (c.829dupG, p.Ala277Glyfs*11) in exon 3 in the *RP2* gene. No retinal disease was known in other family members, assessment of the parental retinal phenotype was not possible, and samples for segregation analysis were not available.

**Fig 7:**
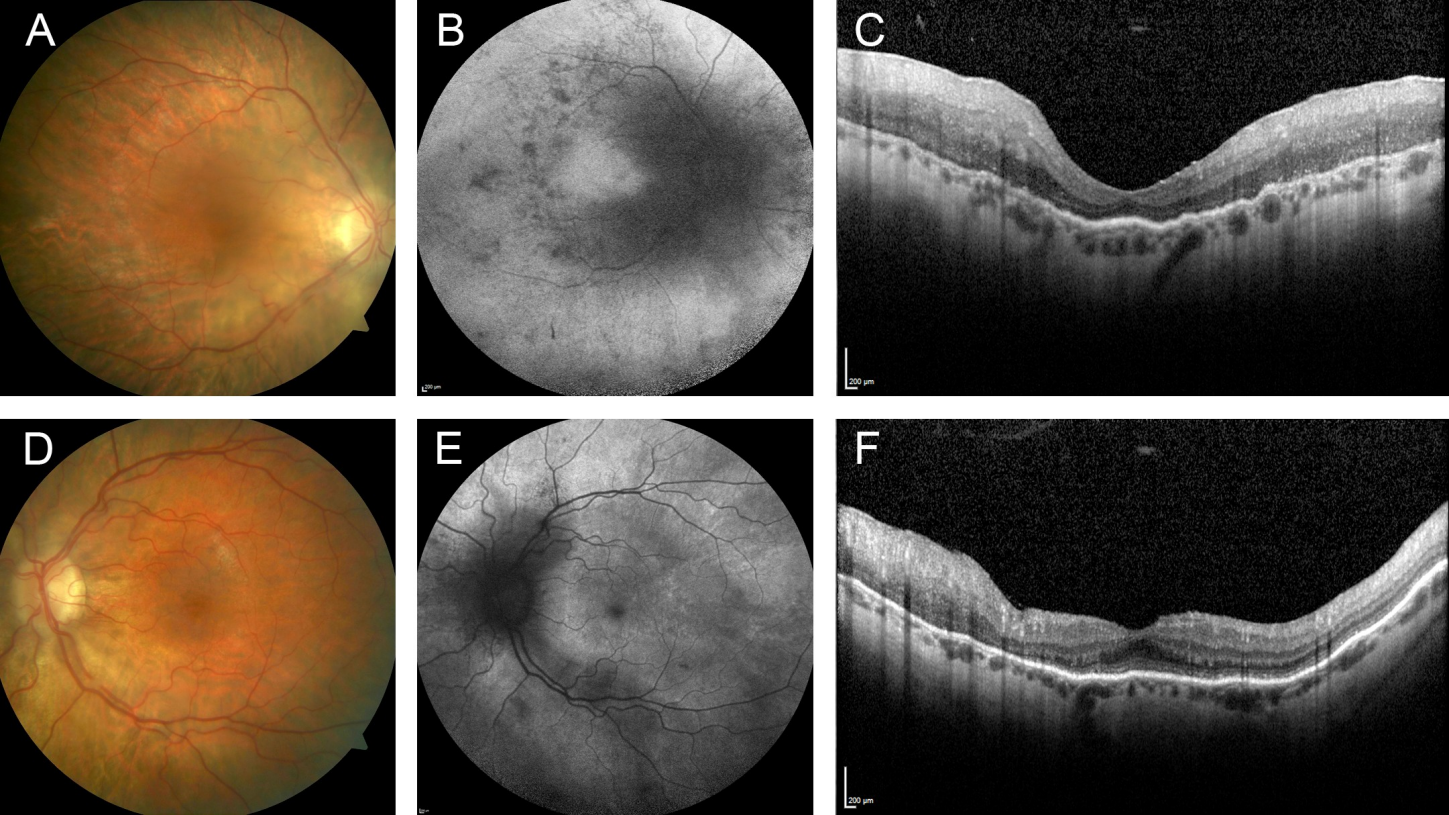
Phenotype of an RP patient with an *RPGR* mutation. A-E right eye, F-J left eye: Fundus color imaging (A, D), fundus autofluorescence with 488 nm excitation light (B, E) and (C, F) spectral-domain optical coherence tomography.

Patient B (*RPGR*): This 39-years-old myopic (spherical equivalent -5 dpt in the right eye and -6,75 dpt in the left eye) woman (#69, S2 Table) with nyctalopia since childhood reported decreasing visual acuity and visual fields since her twenties. Visual acuity was 20/400 in the right eye and 20/63 in the left eye. ERG examination was not tolerated by the patient. Funduscopy revealed changes characteristic for RP including bone spicule pigmentation and attenuated retinal vessels. Fundus AF showed areas of increased and decreased AF in the right eye and a fine pattern of radial lines radiating peripherally from the fovea in the left eye. On OCT imaging, there was widespread thinning of the photoreceptor layer in both eyes with foveal sparing in the left eye. Furthermore, OCT imaging revealed thickening of the inner retina mainly around the optic disc, which was more obvious in the right than in the left eye (Fig 7). We identified a heterozygous four-base-pair deletion (c.2442_2445del, p.Gly817Lysfs*2) in *ORF15* of *RPGR*. None of the patient’s parents had a history suggestive for retinal disease, and examination of the mother including AF-recordings showed no characteristics for a carrier state of X-linked RP. Genetic analysis of the mother revealed wild-type *RPGR* alleles, indicating a *de novo RPGR* mutation in the index patient.

**Fig 8:**
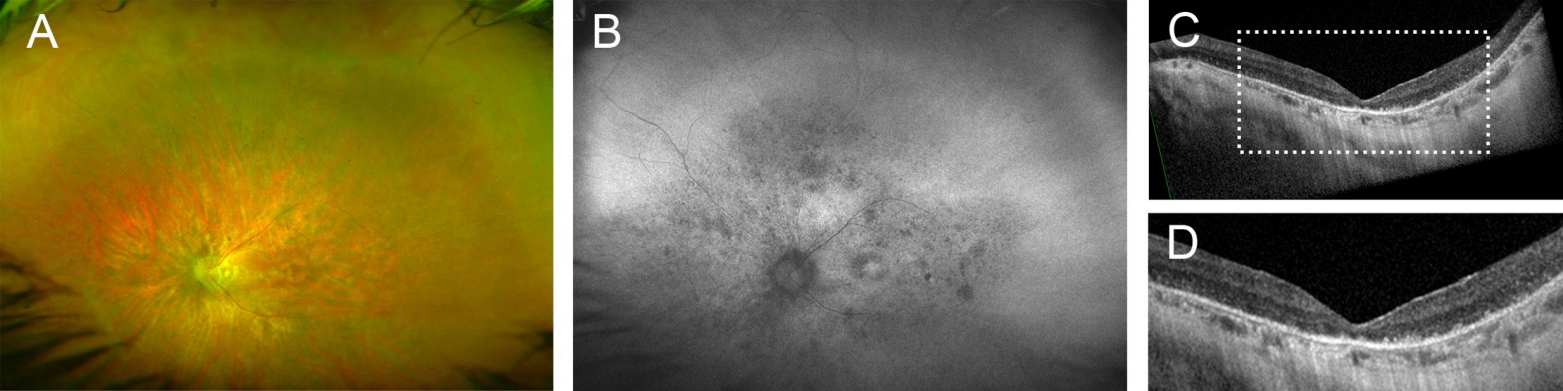
Phenotype of an RP patient with an *RPGR* mutation. (A) widefield fundus AF, (B) widefield false-color image and (C, D) spectral-domain optical coherence tomography. Only one eye is shown due to high symmetry between eyes.

Patient C (*RPGR*): This 52-years-old myopic (spherical equivalent approximately -4 dpt in both eyes) woman (#76, S2 Table) with difficulty in seeing in the dark since childhood reported a progressive reduction of visual acuity over the past 10–20 years. Visual acuity was above 20/50 in adolescence, 20/400-20/200 around the age of 40 years and now 20/800. ERG examination showed no detectable responses. Funduscopy revealed changes characteristic for RP in both eyes. There was symmetric and widespread thinning of the photoreceptor layer on OCT imaging (Fig 8). Although fundus AF imaging showed no sign for an X-linked carrier state, we identified a heterozygous two-base-pair deletion (c.2405_2406delAG, p.Glu802Glyfs*32) in *ORF15* of *RPGR*. The patient’s maternal great-uncle was visually impaired, and her maternal great-grandfather was blind, compatible with autosomal dominant inheritance with reduced penetrance. The mother of the patient died at the age of 50 years and had no visual problems.

Female carriers of X-linked RP consistently have peripheral retinal pigment epithelial atrophy.[62] Most carriers may experience mild or moderate reduction of visual function, with a minority becoming legally blind.[63] Although rare, severe RP may occur in female carriers of X-linked RP and simulate autosomal dominant inheritance[15, 31, 62, 64–66], as in Patient C. Comprehensive genetic testing has been shown to detect mutations in *RPGR* or *RP2* in cohorts assumed to have autosomal dominant RP, leading to a genetic re-classification of those families.[15, 31] However, to the best of our knowledge, a sporadic female RP patient diagnosed with X-linked RP has only been reported once.[5] Of note, parental testing for the *RPGR* mutation of patient B indicated that it occurred *de novo*.

Herein, severe manifestations of X-linked RP were found in two sporadic female carriers and in one patient with a family history suggesting autosomal dominant inheritance with variable expressivity. Two of these three patients revealed very subtle signs for X-linked RP which may easily be missed by standard clinical examinations. This includes asymmetry between eyes which has been reported as a typical sign in X-linked RP-carriers,[67] although marked asymmetry was specifically mentioned in only one out of 61 carriers.[62] Another peculiar finding on fundus AF imaging in female carriers of X-linked RP is a radial pattern extending peripherally from the fovea,[68–70] as observed in a more or less subtle form in the less affected eye of Patients A and B. Moreover, peripapillary thickening of inner retinal layers as observed in patient 2 has been reported in males with X-linked RP.[71, 72] Thus, a subtle X-linked RP carrier phenotype was present in the less severely affected eye in two patients, but not in the third patient who presented with a progressed bilateral disease stage. Unbiased molecular genetic testing eventually revealed the correct diagnosis in these female patients with sporadic RP or with a family history suggestive for autosomal dominant inheritance with variable expressivity.

## Conclusion

In summary, this study demonstrates the enormous genetic heterogeneity of RP and a high detection rate of disease-causing mutations in RP patients using targeted NGS. Given the continuous decline of NGS costs, initial Sanger sequencing of likely disease-causing genes does not appear necessary anymore. Novel genotype-phenotype correlations (*CRX*, *RPGRIP1*) were uncovered, and recently reported novel correlations were confirmed (*CEP290*, *MFSD8*). *PRPF31* and *RPGR* mutations revealed unexpected inheritance modes in a subset of patients. Massively parallel sequencing of all known retinal dystrophy genes is a valuable diagnostic approach in RP patients.

## Supporting information

S1 Table RP panel design. RP genes included in the NGS panels at the respective time of analysis. Importantly, the NGS panels also contained genes for clinically overlapping conditions such as cone/cone-rod dystrophies, Leber’s congenital amaurosis and syndromes with retinal dystrophies, allowing for an extended genetic assessment if no mutation was found in genes previously associated with RP. +: added genes, -: removed genes (compared to the respective previous panel version). arRP, X-linked RP: I (8/10), 32 genes; II (8/13), 63 genes; III (4/15), 73 genes; IV (8/15), 74 genes; V (1/16), 85 genes. adRP: I (8/10), 23 genes; II (8/13), 21 genes; III (4/15), 25 genes; IV (1/16), 31 genes.
(DOCX)

S2 Table Mutations identified in this study. (DOCX)

S3 Table Genes and respective NM numbers identified in this study. (DOCX)

S4 Table Identified novel mutations including allele frequencies and in-silico prediction. (DOCX)
