# Supplementary material for: Next-generation sequencing identifies unexpected genotype-phenotype correlations in patients with retinitis pigmentosa
Source: PLoS One. 2018 Dec 13;13(12):e0207958. doi: 10.1371/journal.pone.0207958 (PMC6292620; doi:10.1371/journal.pone.0207958)
Supplement: S1 Table — RP genes included in the NGS panels at the respective time of analysis. Importantly, the NGS panels also contained genes for clinically overlapping conditions such as cone/cone-rod dystrophies, Leber’s congenital amaurosis and syndromes with retinal dystrophies, allowing for an extended genetic assessment if no mutation was found in genes previously associated with RP. +: added genes, -: removed genes (compared to the respective previous panel version). arRP, X-linked RP: I (8/10), 32 genes; II (8/13), 63 genes; III (4/15), 73 genes; IV (8/15), 74 genes; V (1/16), 85 genes. adRP: I (8/10), 23 genes; II (8/13), 21 genes; III (4/15), 25 genes; IV (1/16), 31 genes. (DOCX) [file pone.0207958.s001.docx]

**arRP, X-linked RP**

**I (8/10)** *ABCA4, ARL6, BEST1, C2ORF71, CDHR1, CERKL, CNGA1, CNGB1, CRB1, EYS, IDH3B, LRAT, MERTK, NR2E3, NRL, PDE6A, PDE6B, PDE6G, PRCD, PROM1, RBP3, RDH5, RGR, RHO, RLBP1, RP1, RP2, RPE65, SAG, SPATA7, TTC8, TULP1*

**II (8/13) +** *ACACB, BBS2, C8ORF37, CLRN1, CYP4V2, DHDDS, DHX38, EMC1, FAM161A, FLVCR1, GNPTG, GPR125, GRID2, IMPG2, KIAA1549, MAK, MPDZ, MTTP, MVK, NEK2, PLA2G5, PRPH2, RBP4, RDH12, RERG, RHBDD2, TPPA, USH1C, USH2A, WDR19, ZNF513*

**III (4/15) +** *ABHD12, ARL2BP, CC2D2A, IFT140, IFT172, KIZ, NEUROD1, PNPLA6, RDH11, SLC7A14, TUB*

**-** *RERG*

**IV (8/15) +** *BBS4*

**V (1/16) +** *ADGRA3, ADIPOR1, AGBL5, ASRGL1, CDH16, DNAJC17, GNS, HGSNAT, LAMA1, TRNT1, ZNF408*

**adRP**

**I (8/10)** *BEST1, CA4, CRX, FSCN2, GUCA1B, IMPDH1, KLHL7, NR2E3, NRL, PRPF3, PRPF31, PRPF8, PRPH2, RDH12, RGR, RHO, ROM1, RP1, RP9, RPE65, SEMA4A, SNRNP200, TOPORS*

**II (8/13) -** *RGR, RH*

**III (4/15) +** *HK1, PRPF4, TEAD1, CAPN5, ITM2B*

**-** *RP9*

**IV (1/16) +** *CTNNA1, KIF5A, MIR204, OR2W3, SPP2, TEAD1*
