# Supplementary material for: Next-generation sequencing identifies unexpected genotype-phenotype correlations in patients with retinitis pigmentosa
Source: PLoS One. 2018 Dec 13;13(12):e0207958. doi: 10.1371/journal.pone.0207958 (PMC6292620; doi:10.1371/journal.pone.0207958)
Supplement: S2 Table — (DOCX) [file pone.0207958.s002.docx]

| **ID (#)** | **Panel** | **Gender**  **(m/f)** | **Gene** | **Zygosity** | **Exon/Intron**  **(IVS)** | **Nucleotide** | **Protein** | **ACMG variant**  **classification**  **standards** | **rs number** | **Segregation**  **analysis** | **Reference** |
| --- | --- | --- | --- | --- | --- | --- | --- | --- | --- | --- | --- |
| **Autosomal recessive** | | | | | | | | | | | |
| 1 | Sanger | m | *EYS* | Homozygous | Exon 32 | c.6544_6547delAACA | p.Asn2182Valfs*2 | Pathogenic | none | no | novel |
| 2 | I | f | *EYS* | Heterozygous | Exon 26 Exon 26 Exon 35 | c.4462_4469dupAGCCCCTC c.4597_4613delTCAAGCAACCAGAGACT  duplication of exon 35 | p.Met1491Alafs*12 p.Ser1533Hisfs*9 p.(?) | Likely pathogenic  Likely pathogenic  Uncertain significance | none  none  none | no | novel novel novel |
| 3 | Sanger | m | *EYS* | Heterozygous | Exon 33 Exon 40 | c.6714delT c.7810C>T | p.Ile2239Serfs*17 p.Arg2640Cys | Pathogenic  Likely pathogenic | rs752953889  rs1015857165 | no | [[1](#_ENREF_1)]  [[2](#_ENREF_2)] |
| 4 | Sanger | f | *EYS* | Heterozygous | Exon 26  Exon 1 | c.4045C>T  deletion of exon 1 | p.Arg1349*  p.(?) | Pathogenic  Uncertain significance | none  none | yes | [[3](#_ENREF_3)]  [[4](#_ENREF_4)] |
| 5 | Sanger | m | *EYS* | Heterozygous | Exon 26 Exon 43 | c.4350_4356delTATAGCT c.8598delA | p.Ile1451Profs*3 p.Gly2867Valfs*5 | Pathogenic  Pathogenic | rs761238771  rs1050742628 | no | [[5](#_ENREF_5)] novel |
| 6 | II | m | *EYS* | Homozygous | Exon 28 | c.5927G>T | p.Arg1976Met | Uncertain significance | none | no | novel |
| 7 | Sanger | m | *EYS* | Homozygous | Intron 8 | c.1299+3A>C | p.Arg437* | Pathogenic | none | no | [[2](#_ENREF_2)] |
| 8 | Sanger | f | *EYS* | Heterozygous | Exon 43  Exon 29 | c.8648_8655delCATGCAGA  duplication of exon 29 | p.Thr2883Lysfs*4  p.(?) | Pathogenic  Uncertain significance | rs528919874  none | no | [[5](#_ENREF_5)]  [[4](#_ENREF_4),[6](#_ENREF_6)] |
| 9 | Sanger | f | *EYS* | Heterozygous | Exon 43  Exon 29 | c.8793_8796delATCA  duplication of exon 29 | p.Gln2931Hisfs*43  p.(?) | Pathogenic  Uncertain significance | none  none | no | novel  [[4](#_ENREF_4),[6](#_ENREF_6)] |
| 10 | Sanger | m | *EYS* | Homozygous | Exon 34 | deletion of exon 34 | p.(?) | Likely pathogenic | none | no | [[4](#_ENREF_4),[7](#_ENREF_7)] |
| 11 | III | m | *USH2A* | Heterozygous | Intron 19 Exon 61 | c.4251+3A>G c.11864G>A | Splice  p.Trp3955* | Uncertain significance  Pathogenic | none  rs111033364 | no | novel [[8](#_ENREF_8)] |
| 12 | III | m | *USH2A* | Heterozygous | Exon 13 Exon 63 | c.2276G>T  c.12575G>A | p.Cys759Phe p.Arg4192His | Likely pathogenic  Uncertain significance | rs80338902  rs199605265 | no | [[9](#_ENREF_9),[10](#_ENREF_10)] [[11](#_ENREF_11),[12](#_ENREF_12)] |
| 13 | IV | f | *USH2A* | Homozygous | Exon 45-47 | deletion of exons 45-47 | p.(?) | Likely pathogenic | none | yes | [[13](#_ENREF_13)] |
| 14 | Sanger | m | *USH2A* | Heterozygous | Exon 13 Exon 22 Exon 63 | c.2299delG c.4714C>T c.13570A>G | p.Glu767Serfs*21 p.Leu1572Phe p.Lys4524Glu | Pathogenic  Uncertain significance  Uncertain significance | rs80338903  rs111033333  none | no | [[14](#_ENREF_14)] [[15](#_ENREF_15)] novel |
| 15 | II | f | *USH2A* | Heterozygous | Exon 13 Exon 22 | c.2276G>T  c.4710delT | p.Cys759Phe p.Phe1570Leufs*5 | Pathogenic  Pathogenic | rs80338902  none | no | [[16](#_ENREF_16)]  novel |
| 16 | IV | m | *USH2A* | Heterozygous | Exon 6  Exon 63 | c.1137delG  c.12575G>A | p.Gln379Hisfs*19  p.Arg4192His | Likely pathogenic  Uncertain significance | none  rs199605265 | yes | novel  [[11](#_ENREF_11),[12](#_ENREF_12)] |
| 17 | V | m | *USH2A* | Heterozygous | Exon 56  Exon 36  Exon 65 | c.10974_10975dupTA  c.6902T>C  c.14219C>A | p.Thr3659Ilefs*16  p.Leu2301Ser  p.Ala4740Asp | Pathogenic  Likely pathogenic  Likely pathogenic | none  rs759494205  rs539192853 | no | novel  [[17](#_ENREF_17)]  [[17](#_ENREF_17)] |
| 18 | II | m | *CRB1* | Heterozygous | Exon 5 Exon 6 | c.1148G>A c.1945G>T | p.Cys383Tyr p.Asp649Tyr | Pathogenic  Likely pathogenic | rs62645754  none | no | [[18](#_ENREF_18),[19](#_ENREF_19)] novel |
| 19 | III | m | *CRB1* | Homozygous | Exon 9 | c.3121A>G | p.Met1041Val | Pathogenic | rs781705903 | yes | novel |
| 20 | I | f | *CRB1* | Heterozygous | Exon 6 Exon 7 | c.2042G>A c.2308G>C | p.Cys681Tyr p.Gly770Arg | Pathogenic  Likely pathogenic | rs62636266  none | yes | [[18](#_ENREF_18)] [[20](#_ENREF_20)] |
| 21 | V | m | *CRB1* | Homozygous | Exon 5 | c.1148G>A | p.Cys383Tyr | Pathogenic | rs62645754 | yes | [[18](#_ENREF_18),[21](#_ENREF_21)] |
| 22 | II | m | *PDE6b* | Homozygous | Exon 1 | c.293G>C | p.Arg98Pro | Likely pathogenic | none | no | novel |
| 23 | Sanger | m | *PDE6b* | Homozygous | Exon 1 | c.299G>A | p.Arg100His | Pathogenic | rs555600300 | no | [[22](#_ENREF_22)] |
| 24 | II | f | *PDE6b* | Homozygous | Exon 1 | c.243delG | p.Arg82Alafs*68 | Likely pathogenic | none | no | [[23](#_ENREF_23)] |
| 25 | II | f | *NR2E3* | Homozygous | Exon 3 | c.309C>A | p.Cys103* | Likely pathogenic | none | no | novel |
| 26 | IV | m | *NR2E3* | Heterozygous | Exon 2 | c.119-2A>C c.227G>A | Splice  p.Arg76Gln | Pathogenic  Uncertain significance | rs2723341  none | yes | [[17](#_ENREF_17)] [[24](#_ENREF_24),[25](#_ENREF_25)] |
| 27 | II | m | *NR2E3* | Homozygous | Exon 8 | c.1049A>G | p.Gln350Arg | Pathogenic | rs756678889 | yes | [[26](#_ENREF_26)] |
| 28 | I | f | *RDH12* | Heterozygous | Exon 5 Exon 9 | c.226G>C c.869T>G | p.Gly76Arg p.Val290Gly | Pathogenic  Pathogenic | rs368489658  rs61740289 | no | [[27](#_ENREF_27)] [[3](#_ENREF_3)] |
| 29 | V | f | *RDH12* | Heterozygous | Exon 7  Exon 7 | c.701G>A  c.806_810delCCCTG | p.R234H  p.A269Gfs*2 | Pathogenic  Pathogenic | rs750636662  rs386834261 | no | [[28](#_ENREF_28),[29](#_ENREF_29)]  [[30](#_ENREF_30),[31](#_ENREF_31)] |
| 30 | II | m | *CDHR1* | Homozygous | Exon 17A | c.2522_2528delTCTCTGA | p.Ile841Serfs*119 | Likely pathogenic | none | yes | [[7](#_ENREF_7),[32](#_ENREF_32)] |
| 31 | II | f | *CNGA1* | Heterozygous | Exon 10 | c.1166C>T c.1280T>C | p.Ser389Phe p.Leu427Pro | Uncertain significance  Uncertain significance | rs62625014  rs748126956 | no | [[33](#_ENREF_33)] novel |
| 32 | III | f | *CNGB1* | Heterozygous | Exon 23 Intron 25 | c.2284C>T c.2492+1G>A | p.Arg762Lys Splice | Pathogenic  Likely pathogenic | none  rs530551814 | yes | [[34](#_ENREF_34),[35](#_ENREF_35)] novel |
| 33 | II | f | *FAM161A* | Homozygous | Exon 3 | c.1567C>T | p.Arg523* | Pathogenic | rs202193201 | no | [[36](#_ENREF_36)] |
| 34 | Sanger | m | *PDE6A* | Heterozygous | Exon 1 Exon 13 Exon 17 | c.304C>A c.1689C>A c.2131G>A | p.Arg102Ser p.His563Gln p.Val711Ile | Likely pathogenic  Likely pathogenic  Uncertain significance | rs141252097  rs776918069  rs764962408 | no | [[11](#_ENREF_11),[37](#_ENREF_37)] novel novel |
| 35 | II | f | *IMPG2* | Homozygous | Exon 13 | c.2143delT | p.Tyr715Thrfs*10 | Likely pathogenic | none | no | novel |
| 36 | II | f | *CEP290* | Heterozygous | Exon 12 Intron 26 | c.982C>T c.2991+1655A>G | p.Gln328* Splice | Pathogenic  Uncertain significance | none  rs281865192 | yes | novel [[3](#_ENREF_3),[38](#_ENREF_38)] |
| 37 | II | m | *MFSD8* | Homozygous | Exon 13 | c.1445G>C | p.Arg482Pro | Uncertain significance | rs547726489 | yes | novel |
| 38 | Sanger | f | *RP1* | Heterozygous | Exon 4 Exon 4 | c.1663C>G c.2029C>T | p.Gln555Glu p.Arg677* | Uncertain significance  Pathogenic | none  rs104894082 | no | novel  [[3](#_ENREF_3)] |
| 39 | II | m | *RPGRIP1* | Homozygous | Exon 19 | deletion of exon 19 | p.(?) | Uncertain significance | none | no | [[39](#_ENREF_39),[40](#_ENREF_40)] |
| 40 | II | m | *RLBP1* | Homozygous | Exon 6 | c.451C>T | p.Arg151Trp | Pathogenic | none | no | [[41](#_ENREF_41)] |
| 41 | IV | f | *C21orf2* | Heterozygous | Exon 1  Intron 4 | c.33_34insAGCTGCACAGCGTGCA  c.374-3A>T | p.Ala12Serfs*60  Splice | Pathogenic  Uncertain significance | rs748531024  rs770721510 | yes | [[7](#_ENREF_7)]  novel |
| 42 | II | f | *PROM1* | Homozygous | Exon 16 | c.1853T>G | p.Leu618Arg | Pathogenic | none | yes | novel |
| 43 | II | f | *ABCA4* | Heterozygous | Exon 12 Exon 21  Exon 13 | c.1622T>C  c.3113C>T  c.1891G>A | p.Leu541Pro p.Ala1038Val p.Gly631Arg | Pathogenic  Likely pathogenic Pathogenic | rs61751392  rs61751374 rs886044730 | yes | [[42](#_ENREF_42),[43](#_ENREF_43)]  [[42](#_ENREF_42),[43](#_ENREF_43)]  [[44](#_ENREF_44)] |
| 44 | II | m | *CERKL* | Heterozygous | Exon 6 Intron 9 | c.847C>T c.1212-3T>A | p.Arg283* Splice | Pathogenic  Uncertain significance | rs121909398 | yes | [[45](#_ENREF_45)] novel |
| 45 | IV | m | *CERKL* | Heterozygous | Exon 1  Exon 2 | c.197_200dupGAGC  deletion of exon 2 | p.Leu68Serfs*15  deletion of exon 2 | Pathogenic  Uncertain significance | none  none | no | novel  novel |
| **Autosomal dominant** | | | | | | | | | | | |
| 46 | I | f | *PRPF31* | Heterozygous | Exon 8 | c.839T>G | p.Val280Gly | Likely pathogenic | none | yes | novel |
| 47 | II | m | *PRPF31* | Heterozygous | Exon 1-3 | deletion of exons 1-3 | deletion of exons 1-3 | Likely pathogenic | none | yes | [[3](#_ENREF_3),[46](#_ENREF_46)] |
| 48 | III | f | *PRPF31* | Heterozygous | Intron 7 | c.698-1G>A | Splice | Pathogenic | none | no | [[47](#_ENREF_47)] |
| 49 | I | f | *PRPF31* | Heterozygous | Exon 1-5 | deletion of exons 1-5 | deletion of exons 1-5 | Pathogenic | none | yes | [[3](#_ENREF_3),[46](#_ENREF_46)] |
| 50 | Sanger | m | *PRPF31* | Heterozygous | Exon 8 | c.816_830delCTACATCTACCACAG | p.Tyr273_Ser277del | Likely pathogenic | none | no | novel |
| 51 | Sanger | f | *PRPF31* | Heterozygous | Intron 6 | c.527+3A>G | IVS6+3A>G | Pathogenic | rs587776590 | no | [[48](#_ENREF_48),[49](#_ENREF_49)] |
| 52 | Sanger | f | *PRPF31* | Heterozygous | Exon 5 | c.330_333delCCAT | p.His111Serfs*86 | Likely pathogenic | none | no | novel |
| 53 | Sanger | f | *PRPF31* | Heterozygous | Exon 3-4 | deletion of exons 1-5 | deletion of exons 1-5 | Likely pathogenic | none | no | [[3](#_ENREF_3),[46](#_ENREF_46)] |
| 54 | I | m | *RHO* | Heterozygous | Intron 4 | c.937-1G>T | Splice | Pathogenic | none | no | [[50](#_ENREF_50)] |
| 55 | Sanger | f | *RHO* | Heterozygous | Exon 3 | c.541G>A | p.Glu181Lys | Pathogenic | rs775557680 | no | [[51](#_ENREF_51),[52](#_ENREF_52)] |
| 56 | Sanger | f | *RHO* | Heterozygous | Exon 1 | c.44A>T | p.Asn15Ile | Likely pathogenic | none | no | [[53](#_ENREF_53),[54](#_ENREF_54),[55](#_ENREF_55)] |
| 57 | Sanger | f | *RHO* | Heterozygous | Exon 5 | c.1028G>A | p.Ser343Asn | Pathogenic | none | no | [[56](#_ENREF_56)] |
| 58 | III | f | *SNRNP200* | Heterozygous | Exon 16 | c.2042G>A | p.Arg681His | Pathogenic | rs527236113 | no | [[57](#_ENREF_57)] |
| 59 | IV | m | *SNRNP200* | Heterozygous | Exon 13 | c.1547G>T | p.Cys516Phe | Likely pathogenic | none | no | novel |
| 60 | IV | m | *SNRNP200* | Heterozygous | Exon 16 | c.2041C>T | p.Arg681Cys | Pathogenic | none | no | [[58](#_ENREF_58),[59](#_ENREF_59)] |
| 61 | I | f | *TOPORS* | Heterozygous | Exon 3 | c.2550_2553delCAGA | p.Asp850Glufs*15 | Pathogenic | none | no | [[3](#_ENREF_3)] |
| 62 | III | f | *TOPORS* | Heterozygous | Exon 3 | c.2515G>T | p.Glu839* | Pathogenic | none | no | [[60](#_ENREF_60)] |
| 63 | III | m | *CRX* | Heterozygous | Exon 3 | c.122G>A | p.Arg41Gln | Pathogenic | rs61748436 | yes | [[61](#_ENREF_61)] |
| 64 | I | m | *NR2E3* | Heterozygous | Exon 2 | c.166G>A | p.Gly56Arg | Pathogenic | rs121912631 | no | [[62](#_ENREF_62),[63](#_ENREF_63)] |
| 65 | IV | f | *PRPF8* | Heterozygous | Exon 40 | c.6446_6462delinsACCACCACACCATG | p.Pro2149_His2154delinsHisHisHisThrMet | Uncertain significance | none | no | novel |
| 66 | IV | f | *PRPH2* | Heterozygous | Exon 1 | c.422A>G | p.Tyr141Cys | Pathogenic | rs61755781 | yes | [[64](#_ENREF_64)] |
| **X-linked** | | | | | | | | | | | |
| 67 | Sanger | m | *RPGR* | Hemizygous | Intron 1 | c.29-1G>T | Splice | Likely pathogenic | none | no | novel |
| 68 | III | f | *RPGR* | Heterozygous | ORF15 | c.2442_2445delAGAG | p.Gly817Lysfs*2 | Likely pathogenic | none | no | [[65](#_ENREF_65)] |
| 69 | ORF15-Amplicon | m | *RPGR* | Hemizygous | ORF15 | c.2452G>T | p.Glu818* | Likely pathogenic | none | no | novel |
| 70 | ORF15-Amplicon | m | *RPGR* | Hemizygous | ORF15 | c.2706_2707delGG | p.Glu903Glyfs*175 | Likely pathogenic | none | no | novel |
| 71 | IV | m | *RPGR* | Hemizygous | Exon 8 | c.917A>C | p.His306Pro | Likely pathogenic | none | no | novel |
| 72 | ORF15-Amplicon | m | *RPGR* | Hemizygous | ORF15 | c.2630delA | p.Glu877Glyfs*212 | Likely pathogenic | none | yes | novel |
| 73 | IV | m | *RPGR* | Hemizygous | Exon 9 | c.1006A>T | p.Asn336Tyr | Likely pathogenic | none | no | novel |
| 74 | ORF15-Amplicon | m | *RPGR* | Hemizygous | ORF15 | c.2426_2427delAG | p.Glu809Glyfs*25 | Likely pathogenic | none | no | [[65](#_ENREF_65)] |
| 75 | III | f | *RPGR* | Heterozygous | ORF15 | c.2405_2406delAG | p.Glu802Glyfs*32 | Likely pathogenic | none | no | [[65](#_ENREF_65)] |
| 76 | IV | m | *RPGR* | Hemizygous | ORF15 | c.3034delG | p.Glu1012Lysfs*77 | Likely pathogenic | none | no | novel |
| 77 | V | m | *RPGR* | Hemizygous | Exon 3 | c.194G>T | p.Gly65Val | Pathogenic | none | no | [[66](#_ENREF_66),[67](#_ENREF_67),[68](#_ENREF_68)] |
| 78 | ORF15-Amplicon | m | *RPGR* | Hemizygous | ORF15 | c.2313_2314delinsC | p.Glu771Aspfs*44 | Pathogenic | none | no | novel |
| 79 | III | f | *RP2* | Heterozygous | Exon 3 | c.829dupG | p.Ala277Glyfs*11 | Likely pathogenic | none | no | novel |
| 80 | I | m | *RP2* | Heterozygous | Exon 2 | c.630_633delTCGT | p.Arg211Phefs*26 | Likely pathogenic | none | no | novel |
| 81 | V | m | *RP2* | Hemizygous | Exon 2 | c.758delT | p.Leu253Glnfs*12 | Pathogenic | none | no | novel |

**References**

1. Collin RW, Littink KW, Klevering BJ, van den Born LI, Koenekoop RK, et al. (2008) Identification of a 2 Mb human ortholog of Drosophila eyes shut/spacemaker that is mutated in patients with retinitis pigmentosa. Am J Hum Genet 83: 594-603.

2. Abd El-Aziz MM, O'Driscoll CA, Kaye RS, Barragan I, El-Ashry MF, et al. (2010) Identification of novel mutations in the ortholog of Drosophila eyes shut gene (EYS) causing autosomal recessive retinitis pigmentosa. Invest Ophthalmol Vis Sci 51: 4266-4272.

3. Eisenberger T, Neuhaus C, Khan AO, Decker C, Preising MN, et al. (2013) Increasing the yield in targeted next-generation sequencing by implicating CNV analysis, non-coding exons and the overall variant load: the example of retinal dystrophies. PLoS One 8: e78496.

4. Pieras JI, Barragan I, Borrego S, Audo I, Gonzalez-Del Pozo M, et al. (2011) Copy-number variations in EYS: a significant event in the appearance of arRP. Invest Ophthalmol Vis Sci 52: 5625-5631.

5. Littink KW, van den Born LI, Koenekoop RK, Collin RW, Zonneveld MN, et al. (2010) Mutations in the EYS gene account for approximately 5% of autosomal recessive retinitis pigmentosa and cause a fairly homogeneous phenotype. Ophthalmology 117: 2026-2033, 2033 e2021-2027.

6. Nishiguchi KM, Tearle RG, Liu YP, Oh EC, Miyake N, et al. (2013) Whole genome sequencing in patients with retinitis pigmentosa reveals pathogenic DNA structural changes and NEK2 as a new disease gene. Proc Natl Acad Sci U S A 110: 16139-16144.

7. Carss KJ, Arno G, Erwood M, Stephens J, Sanchis-Juan A, et al. (2017) Comprehensive Rare Variant Analysis via Whole-Genome Sequencing to Determine the Molecular Pathology of Inherited Retinal Disease. Am J Hum Genet 100: 75-90.

8. Shearer AE, Eppsteiner RW, Booth KT, Ephraim SS, Gurrola J, 2nd, et al. (2014) Utilizing ethnic-specific differences in minor allele frequency to recategorize reported pathogenic deafness variants. Am J Hum Genet 95: 445-453.

9. Rivolta C, Sweklo EA, Berson EL, Dryja TP (2000) Missense mutation in the USH2A gene: association with recessive retinitis pigmentosa without hearing loss. Am J Hum Genet 66: 1975-1978.

10. Zhao L, Wang F, Wang H, Li Y, Alexander S, et al. (2015) Next-generation sequencing-based molecular diagnosis of 82 retinitis pigmentosa probands from Northern Ireland. Hum Genet 134: 217-230.

11. Avila-Fernandez A, Cantalapiedra D, Aller E, Vallespin E, Aguirre-Lamban J, et al. (2010) Mutation analysis of 272 Spanish families affected by autosomal recessive retinitis pigmentosa using a genotyping microarray. Mol Vis 16: 2550-2558.

12. McGee TL, Seyedahmadi BJ, Sweeney MO, Dryja TP, Berson EL (2010) Novel mutations in the long isoform of the USH2A gene in patients with Usher syndrome type II or non-syndromic retinitis pigmentosa. J Med Genet 47: 499-506.

13. Baux D, Blanchet C, Hamel C, Meunier I, Larrieu L, et al. (2014) Enrichment of LOVD-USHbases with 152 USH2A genotypes defines an extensive mutational spectrum and highlights missense hotspots. Hum Mutat 35: 1179-1186.

14. Dreyer B, Tranebjaerg L, Brox V, Rosenberg T, Moller C, et al. (2001) A common ancestral origin of the frequent and widespread 2299delG USH2A mutation. Am J Hum Genet 69: 228-234.

15. Song J, Smaoui N, Ayyagari R, Stiles D, Benhamed S, et al. (2011) High-throughput retina-array for screening 93 genes involved in inherited retinal dystrophy. Invest Ophthalmol Vis Sci 52: 9053-9060.

16. Lenassi E, Vincent A, Li Z, Saihan Z, Coffey AJ, et al. (2015) A detailed clinical and molecular survey of subjects with nonsyndromic USH2A retinopathy reveals an allelic hierarchy of disease-causing variants. Eur J Hum Genet 23: 1318-1327.

17. Glockle N, Kohl S, Mohr J, Scheurenbrand T, Sprecher A, et al. (2014) Panel-based next generation sequencing as a reliable and efficient technique to detect mutations in unselected patients with retinal dystrophies. Eur J Hum Genet 22: 99-104.

18. Lotery AJ, Jacobson SG, Fishman GA, Weleber RG, Fulton AB, et al. (2001) Mutations in the CRB1 gene cause Leber congenital amaurosis. Arch Ophthalmol 119: 415-420.

19. Beryozkin A, Zelinger L, Bandah-Rozenfeld D, Harel A, Strom TA, et al. (2013) Mutations in CRB1 are a relatively common cause of autosomal recessive early-onset retinal degeneration in the Israeli and Palestinian populations. Invest Ophthalmol Vis Sci 54: 2068-2075.

20. Kousal B, Dudakova L, Gaillyova R, Hejtmankova M, Diblik P, et al. (2016) Phenotypic features of CRB1-associated early-onset severe retinal dystrophy and the different molecular approaches to identifying the disease-causing variants. Graefes Arch Clin Exp Ophthalmol 254: 1833-1839.

21. den Hollander AI, ten Brink JB, de Kok YJ, van Soest S, van den Born LI, et al. (1999) Mutations in a human homologue of Drosophila crumbs cause retinitis pigmentosa (RP12). Nat Genet 23: 217-221.

22. Ge Z, Bowles K, Goetz K, Scholl HP, Wang F, et al. (2015) NGS-based Molecular diagnosis of 105 eyeGENE((R)) probands with Retinitis Pigmentosa. Sci Rep 5: 18287.

23. Ullah I, Kabir F, Gottsch CB, Naeem MA, Guru AA, et al. (2016) Mutations in phosphodiesterase 6 identified in familial cases of retinitis pigmentosa. Hum Genome Var 3: 16036.

24. Kanda A, Swaroop A (2009) A comprehensive analysis of sequence variants and putative disease-causing mutations in photoreceptor-specific nuclear receptor NR2E3. Mol Vis 15: 2174-2184.

25. Roduit R, Escher P, Schorderet DF (2009) Mutations in the DNA-binding domain of NR2E3 affect in vivo dimerization and interaction with CRX. PLoS One 4: e7379.

26. Pachydaki SI, Klaver CC, Barbazetto IA, Roy MS, Gouras P, et al. (2009) Phenotypic features of patients with NR2E3 mutations. Arch Ophthalmol 127: 71-75.

27. Aldahmesh MA, Safieh LA, Alkuraya H, Al-Rajhi A, Shamseldin H, et al. (2009) Molecular characterization of retinitis pigmentosa in Saudi Arabia. Mol Vis 15: 2464-2469.

28. Thompson DA, Janecke AR, Lange J, Feathers KL, Hubner CA, et al. (2005) Retinal degeneration associated with RDH12 mutations results from decreased 11-cis retinal synthesis due to disruption of the visual cycle. Hum Mol Genet 14: 3865-3875.

29. Valverde D, Pereiro I, Vallespin E, Ayuso C, Borrego S, et al. (2009) Complexity of phenotype-genotype correlations in Spanish patients with RDH12 mutations. Invest Ophthalmol Vis Sci 50: 1065-1068.

30. Janecke AR, Thompson DA, Utermann G, Becker C, Hubner CA, et al. (2004) Mutations in RDH12 encoding a photoreceptor cell retinol dehydrogenase cause childhood-onset severe retinal dystrophy. Nat Genet 36: 850-854.

31. Wang F, Wang H, Tuan HF, Nguyen DH, Sun V, et al. (2014) Next generation sequencing-based molecular diagnosis of retinitis pigmentosa: identification of a novel genotype-phenotype correlation and clinical refinements. Hum Genet 133: 331-345.

32. Arno G, Hull S, Carss K, Dev-Borman A, Chakarova C, et al. (2016) Reevaluation of the Retinal Dystrophy Due to Recessive Alleles of RGR With the Discovery of a Cis-Acting Mutation in CDHR1. Invest Ophthalmol Vis Sci 57: 4806-4813.

33. Dryja TP, Finn JT, Peng YW, McGee TL, Berson EL, et al. (1995) Mutations in the gene encoding the alpha subunit of the rod cGMP-gated channel in autosomal recessive retinitis pigmentosa. Proc Natl Acad Sci U S A 92: 10177-10181.

34. Azam M, Collin RW, Malik A, Khan MI, Shah ST, et al. (2011) Identification of novel mutations in Pakistani families with autosomal recessive retinitis pigmentosa. Arch Ophthalmol 129: 1377-1378.

35. Bocquet B, Marzouka NA, Hebrard M, Manes G, Senechal A, et al. (2013) Homozygosity mapping in autosomal recessive retinitis pigmentosa families detects novel mutations. Mol Vis 19: 2487-2500.

36. Bandah-Rozenfeld D, Mizrahi-Meissonnier L, Farhy C, Obolensky A, Chowers I, et al. (2010) Homozygosity mapping reveals null mutations in FAM161A as a cause of autosomal-recessive retinitis pigmentosa. Am J Hum Genet 87: 382-391.

37. Dryja TP, Rucinski DE, Chen SH, Berson EL (1999) Frequency of mutations in the gene encoding the alpha subunit of rod cGMP-phosphodiesterase in autosomal recessive retinitis pigmentosa. Invest Ophthalmol Vis Sci 40: 1859-1865.

38. den Hollander AI, Koenekoop RK, Yzer S, Lopez I, Arends ML, et al. (2006) Mutations in the CEP290 (NPHP6) gene are a frequent cause of Leber congenital amaurosis. Am J Hum Genet 79: 556-561.

39. Ellingford JM, Campbell C, Barton S, Bhaskar S, Gupta S, et al. (2017) Validation of copy number variation analysis for next-generation sequencing diagnostics. Eur J Hum Genet 25: 719-724.

40. Thompson JA, De Roach JN, McLaren TL, Montgomery HE, Hoffmann LH, et al. (2017) The genetic profile of Leber congenital amaurosis in an Australian cohort. Mol Genet Genomic Med 5: 652-667.

41. Fishman GA, Roberts MF, Derlacki DJ, Grimsby JL, Yamamoto H, et al. (2004) Novel mutations in the cellular retinaldehyde-binding protein gene (RLBP1) associated with retinitis punctata albescens: evidence of interfamilial genetic heterogeneity and fundus changes in heterozygotes. Arch Ophthalmol 122: 70-75.

42. Webster AR, Heon E, Lotery AJ, Vandenburgh K, Casavant TL, et al. (2001) An analysis of allelic variation in the ABCA4 gene. Invest Ophthalmol Vis Sci 42: 1179-1189.

43. Aguirre-Lamban J, Gonzalez-Aguilera JJ, Riveiro-Alvarez R, Cantalapiedra D, Avila-Fernandez A, et al. (2011) Further associations between mutations and polymorphisms in the ABCA4 gene: clinical implication of allelic variants and their role as protector/risk factors. Invest Ophthalmol Vis Sci 52: 6206-6212.

44. Schulz HL, Grassmann F, Kellner U, Spital G, Ruther K, et al. (2017) Mutation Spectrum of the ABCA4 Gene in 335 Stargardt Disease Patients From a Multicenter German Cohort-Impact of Selected Deep Intronic Variants and Common SNPs. Invest Ophthalmol Vis Sci 58: 394-403.

45. Tuson M, Marfany G, Gonzalez-Duarte R (2004) Mutation of CERKL, a novel human ceramide kinase gene, causes autosomal recessive retinitis pigmentosa (RP26). Am J Hum Genet 74: 128-138.

46. Almoguera B, Li J, Fernandez-San Jose P, Liu Y, March M, et al. (2015) Application of Whole Exome Sequencing in Six Families with an Initial Diagnosis of Autosomal Dominant Retinitis Pigmentosa: Lessons Learned. PLoS One 10: e0133624.

47. Roberts L, Ratnapriya R, du Plessis M, Chaitankar V, Ramesar RS, et al. (2016) Molecular Diagnosis of Inherited Retinal Diseases in Indigenous African Populations by Whole-Exome Sequencing. Invest Ophthalmol Vis Sci 57: 6374-6381.

48. Vithana EN, Abu-Safieh L, Allen MJ, Carey A, Papaioannou M, et al. (2001) A human homolog of yeast pre-mRNA splicing gene, PRP31, underlies autosomal dominant retinitis pigmentosa on chromosome 19q13.4 (RP11). Mol Cell 8: 375-381.

49. Waseem NH, Vaclavik V, Webster A, Jenkins SA, Bird AC, et al. (2007) Mutations in the gene coding for the pre-mRNA splicing factor, PRPF31, in patients with autosomal dominant retinitis pigmentosa. Invest Ophthalmol Vis Sci 48: 1330-1334.

50. Reig C, Alvarez AI, Tejada I, Molina M, Arostegui E, et al. (1996) New mutation in the 3'-acceptor splice site of intron 4 in the rhodopsin gene associated with autosomal dominant retinitis pigmentosa in a Basque family. Hum Mutat 8: 93-94.

51. Blanco-Kelly F, Garcia-Hoyos M, Corton M, Avila-Fernandez A, Riveiro-Alvarez R, et al. (2012) Genotyping microarray: mutation screening in Spanish families with autosomal dominant retinitis pigmentosa. Mol Vis 18: 1478-1483.

52. Dryja TP, Hahn LB, Cowley GS, McGee TL, Berson EL (1991) Mutation spectrum of the rhodopsin gene among patients with autosomal dominant retinitis pigmentosa. Proc Natl Acad Sci U S A 88: 9370-9374.

53. Kranich H, Bartkowski S, Denton MJ, Krey S, Dickinson P, et al. (1993) Autosomal dominant 'sector' retinitis pigmentosa due to a point mutation predicting an Asn-15-Ser substitution of rhodopsin. Hum Mol Genet 2: 813-814.

54. Opefi CA, South K, Reynolds CA, Smith SO, Reeves PJ (2013) Retinitis pigmentosa mutants provide insight into the role of the N-terminal cap in rhodopsin folding, structure, and function. J Biol Chem 288: 33912-33926.

55. Rakoczy EP, Kiel C, McKeone R, Stricher F, Serrano L (2011) Analysis of disease-linked rhodopsin mutations based on structure, function, and protein stability calculations. J Mol Biol 405: 584-606.

56. Van Cauwenbergh C, Coppieters F, Roels D, De Jaegere S, Flipts H, et al. (2017) Mutations in Splicing Factor Genes Are a Major Cause of Autosomal Dominant Retinitis Pigmentosa in Belgian Families. PLoS One 12: e0170038.

57. de Castro-Miro M, Pomares E, Lores-Motta L, Tonda R, Dopazo J, et al. (2014) Combined genetic and high-throughput strategies for molecular diagnosis of inherited retinal dystrophies. PLoS One 9: e88410.

58. Benaglio P, McGee TL, Capelli LP, Harper S, Berson EL, et al. (2011) Next generation sequencing of pooled samples reveals new SNRNP200 mutations associated with retinitis pigmentosa. Hum Mutat 32: E2246-2258.

59. Bowne SJ, Sullivan LS, Avery CE, Sasser EM, Roorda A, et al. (2013) Mutations in the small nuclear riboprotein 200 kDa gene (SNRNP200) cause 1.6% of autosomal dominant retinitis pigmentosa. Mol Vis 19: 2407-2417.

60. Knowles MR, Ostrowski LE, Loges NT, Hurd T, Leigh MW, et al. (2013) Mutations in SPAG1 cause primary ciliary dyskinesia associated with defective outer and inner dynein arms. Am J Hum Genet 93: 711-720.

61. Swain PK, Chen S, Wang QL, Affatigato LM, Coats CL, et al. (1997) Mutations in the cone-rod homeobox gene are associated with the cone-rod dystrophy photoreceptor degeneration. Neuron 19: 1329-1336.

62. Coppieters F, Leroy BP, Beysen D, Hellemans J, De Bosscher K, et al. (2007) Recurrent mutation in the first zinc finger of the orphan nuclear receptor NR2E3 causes autosomal dominant retinitis pigmentosa. Am J Hum Genet 81: 147-157.

63. Blanco-Kelly F, Garcia Hoyos M, Lopez Martinez MA, Lopez-Molina MI, Riveiro-Alvarez R, et al. (2016) Dominant Retinitis Pigmentosa, p.Gly56Arg Mutation in NR2E3: Phenotype in a Large Cohort of 24 Cases. PLoS One 11: e0149473.

64. Sohocki MM, Daiger SP, Bowne SJ, Rodriquez JA, Northrup H, et al. (2001) Prevalence of mutations causing retinitis pigmentosa and other inherited retinopathies. Hum Mutat 17: 42-51.

65. Vervoort R, Lennon A, Bird AC, Tulloch B, Axton R, et al. (2000) Mutational hot spot within a new RPGR exon in X-linked retinitis pigmentosa. Nat Genet 25: 462-466.

66. Fahim AT, Bowne SJ, Sullivan LS, Webb KD, Williams JT, et al. (2011) Allelic heterogeneity and genetic modifier loci contribute to clinical variation in males with X-linked retinitis pigmentosa due to RPGR mutations. PLoS One 6: e23021.

67. Bowne SJ, Sullivan LS, Koboldt DC, Ding L, Fulton R, et al. (2011) Identification of disease-causing mutations in autosomal dominant retinitis pigmentosa (adRP) using next-generation DNA sequencing. Invest Ophthalmol Vis Sci 52: 494-503.

68. Churchill JD, Bowne SJ, Sullivan LS, Lewis RA, Wheaton DK, et al. (2013) Mutations in the X-linked retinitis pigmentosa genes RPGR and RP2 found in 8.5% of families with a provisional diagnosis of autosomal dominant retinitis pigmentosa. Invest Ophthalmol Vis Sci 54: 1411-1416.
