# Supplementary material for: Next-generation sequencing identifies unexpected genotype-phenotype correlations in patients with retinitis pigmentosa
Source: PLoS One. 2018 Dec 13;13(12):e0207958. doi: 10.1371/journal.pone.0207958 (PMC6292620; doi:10.1371/journal.pone.0207958)
Supplement: S3 Table — (DOCX) [file pone.0207958.s003.docx]

| **Gene** | **NM number** |
| --- | --- |
|  |  |
| *EYS* | NM_001142800.1 |
| *USH2A* | NM_206933.2 |
| *CRB1* | NM_201253.2 |
| *PDE6b* | NM_000283.3 |
| *NR2E3* | NM_014249.3 |
| *RDH12* | NM_152443.2 |
| *CDHR1* | NM_033100.3 |
| *CNGA1* | NM_001142564 |
| *CNGB1* | NM_001297.4 |
| *FAM161A* | NM_032180.2 |
| *PDE6A* | NM_000440.2 |
| *IMPG2* | NM_016247.3 |
| *CEP290* | NM_025114.3 |
| *MFSD8* | NM_152778.2 |
| *RP1* | NM_006269.1 |
| *RPGRIP1* | NM_020366.3 |
| *RLBP1* | NM_000326.4 |
| *C21orf2* | NM_004928 |
| *PROM1* | NM_006017.2 |
| *ABCA4* | NM_000350.2 |
| *CERKL* | NM_001030311.2 |
| *PRPF31* | NM_015629.3 |
| *RHO* | NM_000539.3 |
| *SNRNP200* | NM_014014.4 |
| *TOPORS* | NM_005802.4 |
| *CRX* | NM_000554.4 |
| *NR2E3* | NM_014249.3 |
| *PRPF8* | NM_006445.3 |
| *PRPH2* | NM_000322.4 |
| *RPGR* | NM_000328.2 |
| *RPGR ORF15* | NM_001034853 |
| *RP2* | NM_006915.2 |
