# Supplementary material for: Next-generation sequencing identifies unexpected genotype-phenotype correlations in patients with retinitis pigmentosa
Source: PLoS One. 2018 Dec 13;13(12):e0207958. doi: 10.1371/journal.pone.0207958 (PMC6292620; doi:10.1371/journal.pone.0207958)
Supplement: S4 Table — (DOCX) [file pone.0207958.s004.docx]

| **ID (#)** | **Gene** | **Exon/Intron**  **(IVS)** | **Nucleotide** | **Protein** | **gnomAD (allele frequency [%])** | **Functional prediction** | **Conservation prediction** | **Splice site prediction** |
| --- | --- | --- | --- | --- | --- | --- | --- | --- |
| **Missense and splice variants** | | | | | | | | |
| 6 | *EYS* | Exon 28 | c.5927G>T | p.Arg1976Met | n.a. | 4/9 | 3/6 | 2/2 |
| 11 | *USH2A* | Intron 19 | c.4251+3A>G | p.? (Splice donor SNV) | 0.0032 | 0/0 | 0/0 | 2/2 |
| 14 | *USH2A* | Exon 63 | c.13570A>G | p.Lys4524Glu | 0.00041 | 2/9 | 3/6 | n.a. |
| 18 | *CRB1* | Exon 6 | c.1945G>T | p.Asp649Tyr | n.a. | 7/9 | 4/6 | n.a. |
| 19 | *CRB1* | Exon 9 | c.3121A>G | p.Met1041Val | 0.00041 | 7/9 | 3/6 | n.a. |
| 22 | *PDE6B* | Exon 1 | c.293G>C | p.Arg98Pro | n.a. | 8/10 | 5/6 | n.a. |
| 31 | *CNGA1* | Exon 10 | c.1280T>C | p.Leu427Pro | 0.00041 | 10/10 | 5/6 | n.a. |
| 32 | *CNGB1* | Intron 25 | c.2492+1G>A | p.? (Splice donor SNV) | n.a. | 0/0 | 0/0 | 2/2 |
| 34 | *PDE6A* | Exon 13  Exon 17 | c.1689C>A  c.2131G>A | p.His563Gln  p.Val711Ile | 0.00081  0.0018 | 10/10  3/10 | 3/6  3/6 | n.a.  n.a. |
| 37 | *MFSD8* | Exon 13 | c.1445G>C | p.Arg482Pro | 0.00041 | 7/10 | 5/6 | n.a. |
| 38 | *RP1* | Exon 4 | c.1663C>G | p.Gln555Glu | n.a. | 6/10 | 2/6 | n.a. |
| 41 | *C21orf2* | Intron 4 | c.374-3A>T | p.? (Splice acceptor SNV) | n.a. | 0/0 | 0/0 | 0/2 |
| 42 | *PROM1* | Exon 16 | c.1853T>G | p.Leu618Arg | n.a. | 9/10 | 4/6 | n.a. |
| 44 | *CERKL* | Intron 9 | c.1212-3T>A | p.? (Splice acceptor SNV) | n.a. | 0/0 | 0/0 | 0/2 |
| 46 | *PRPF31* | Exon 8 | c.839T>G | p.Val280Gly | n.a. | 10/10 | 5/6 | n.a. |
| 59 | *SNRNP200* | Exon 13 | c.1547G>T | p.Cys516Phe | n.a. | 7/10 | 5/6 | n.a. |
| 67 | *RPGR* | Intron 1 | c.29-1G>T | p.? (Splice acceptor SNV) | n.a. | 0/0 | 0/0 | 2/2 |
| 71 | *RPGR* | Exon 8 | c.917A>C | p.His306Pro | n.a. | 10/10 | 5/6 | n.a. |
| 73 | *RPGR* | Exon 9 | c.1006A>T | p.Asn336Tyr | n.a. | 8/10 | 5/6 | n.a. |
| **Nonsense and frameshift variants, large deletions** | | | | | | | | |
| 1 | *EYS* | Exon 32 | c.6544_6547delAACA | p.Asn2182Valfs*2 | n.a. |  |  |  |
| 2 | *EYS* | Exon 26 Exon 26 Exon 35 | c.4462_4469dupAGCCCCTC c.4597_4613delTCAAGCAACCAGAGACT  duplication of exon 35 | p.Met1491Alafs*12 p.Ser1533Hisfs*9 p.(?) | n.a.  n.a.  n.a. |  |  |  |
| 5 | *EYS* | Exon 43 | c.8598delA | p.Gly2867Valfs*5 | n.a. |  |  |  |
| 9 | *EYS* | Exon 43 | c.8793_8796delATCA | p.Gln2931Hisfs*43 | n.a. |  |  |  |
| 15 | *USH2A* | Exon 22 | c.4710delT | p.Phe1570Leufs*5 | n.a. |  |  |  |
| 16 | *USH2A* | Exon 6 | c.1137delG | p.Gln379Hisfs*19 | n.a. |  |  |  |
| 17 | *USH2A* | Exon 56 | c.10974_10975dupTA | p.Thr3659Ilefs*16 | n.a. |  |  |  |
| 25 | *NR2E3* | Exon 3 | c.309C>A | p.Cys103* | 0.0017 |  |  |  |
| 35 | *IMPG2* | Exon 13 | c.2143delT | p.Tyr715Thrfs*10 | n.a. |  |  |  |
| 36 | *CEP290* | Exon 12 | c.982C>T | p.Gln328* | n.a. |  |  |  |
| 45 | *CERKL* | Exon 1  Exon 2 | c.197_200dupGAGC  deletion of exon 2 | p.Leu68Serfs*15  p.(?) | 0.00043  n.a. |  |  |  |
| 50 | *PRPF31* | Exon 8 | c.816_830delCTACATCTACCACAG | p.Tyr273_Ser277del | n.a. |  |  |  |
| 52 | *PRPF31* | Exon 5 | c.330_333delCCAT | p.His111Serfs*86 | n.a. |  |  |  |
| 65 | *PRPF8* | Exon 40 | c.6446_6462delinsACCACCACACCATG | p.Pro2149_His2154delinsHisHisHisThrMet | n.a. |  |  |  |
| 69 | *RPGR* | ORF15 | c.2452G>T | p.Glu818* | n.a. |  |  |  |
| 70 | *RPGR* | ORF15 | c.2706_2707delGG | p.Glu903Glyfs*175 | n.a. |  |  |  |
| 72 | *RPGR* | ORF15 | c.2630delA | p.Glu877Glyfs*212 | n.a. |  |  |  |
| 76 | *RPGR* | ORF15 | c.3034delG | p.Glu1012Lysfs*77 | n.a. |  |  |  |
| 78 | *RPGR* | ORF15 | c.2313_2314delinsC | p.Glu771Aspfs*44 | n.a. |  |  |  |
| 79 | *RP2* | Exon 3 | c.829dupG | p.Ala277Glyfs*11 | n.a. |  |  |  |
| 80 | *RP2* | Exon 2 | c.630_633delTCGT | p.Arg211Phefs*26 | n.a. |  |  |  |
| 81 | *RP2* | Exon 2 | c.758delT | p.Leu253Glnfs*12 | n.a. |  |  |  |

**Functional predictions:** Functional predictions by SIFT, PolyPhen2, MutationTaster, MutationAssessor, FATHMM, LRT, VEST, CADD, PROVEAN and DANN

**Conservation predictions:** Assessment of conservation by PhyloP, GERP++, PhastCons, SiPhy, Grantham Distance and BLOSUM62

**Splice Site Prediction:** AdaBoost and Random Forest

n.a. = not available
